# Meeting Abstracts for the Society for Simulation in Europe 2024

**DOI:** 10.1186/s41077-024-00287-2

**Published:** 2024-06-17

## O1. Anaesthesiology Trainees Simulation-based Assessment (ATSA)-Study: Assessing Anaesthesiology trainees in Germany and Spain: a pilot study

### Gerard Sergi Angeles Fite^1^, Sara García Ballester^2^, Federico Migliorelli Falcone^3^, Lidia Gómez López^3^, Raquel Bergé, Manuel López-Baamonde^3^, Cristina Ibañez Esteve^3^, Juan Manuel PERDOMO^3^, Tomás Cuñat^3^, Johannes Schäfer^4^, Christopher Neuhaus^4^, Albert Carramiñana^3^, Beatriz Tena^3^, Miriam Fiore Panzeri^3^, Ricard Valero^3^

#### ^1^Universitätsspital Bern (University Hospital of Bern), ^2^Bellvitge University Hospital, ^3^Hospital Clínic de Barcelona, ^4^Universitätsklinikum Heidelberg

*Advances in Simulation 2024*, **9(1):** O1

***Introduction***

Despite efforts of the European Anaesthesiology organisations on harmonizing postgraduate training (1,2), by establishing a common European curriculum (3) and a European end-of-training examination (4), European training programs differ greatly (1,5,6). Medical simulations are wide used in Anaesthesiology training (1,5). Specific tools enable the assessment of residents’ skills in simulated scenarios (7–11), but its use in assessment of Anaesthesiology residents from different training programs across Europe is not well established yet (1,3,5).

The objective of this study was to compare Anaesthesiology residents from two different training systems, combining knowledge evaluation with simulation-based assessment of clinical and non-technical skills.

***Methods***

We performed an observational simulation-based study with anaesthesiology residents in 3rd and 4th year from university hospitals in Germany and Spain. The individual evaluation of each resident consisted of: a) a multiple-choice test (MCQ) based on the European end-of-training examination (4), b) an assessment of clinical skills in two simulated scenarios using a specific scoring-grid based on current guidelines and adapted from existing tools (7,8), and c) an assessment of non-technical skills in two simulated scenarios using a well-validated tool (9). Two scenarios were specifically designed to assess core competencies from the European curriculum (3). The performance of each participant was video-recorded. The assessment of clinical and non-technical skills was performed by two evaluators. Data and characteristics of the participants and their training programs were collected in a survey.

Data are presented as medians (with interquartile ranges). For both clinical and non-technical skills, the mean values from the scores given by each evaluator were used. The results for each group were compared using Wilcoxon Rank-Sum Test.

***Results & Discussion***

We enrolled 43 Anaesthesiology residents, 19 in Spain and 24 in Germany, 23 in 3rd and 20 in 4th year of training in anaesthesiology. The characteristics of the participants were similar in both groups. Results are shown in table 1. The knowledge test, the clinical skills assessment and the non-technical skills assessment were able to distinguish between 3rd and 4th year residents, but there were no significant differences in none of the assessments between the Spanish and the German cohort. In conclusion, the multidimensional assessment model used in our study was able to discriminate between 3rd and 4th year residents in both cohorts, demonstrating validity, but did not show differences among residents from two different training systems.

***Keywords***

Anaesthesiology, Post-graduate medical education, Post-graduate assessment

***References***

1. Van Gessel EF, Ostergard HT, Niemi-Murola L. Harmonisation of anaesthesiology training in Europe. Best Pract ResClin Anaesthesiol. 2012;26(1):55–67.
2. Shorten GD, De Robertis E, Goldik Z, Kietaibl S, Niemi-Murola L, Sabelnikovs O. European Section/Board ofAnaesthesiology/European Society of Anaesthesiology consensus statement on competency-based education and training in anaesthesiology. Eur J Anaesthesiol EJA. 2020;37(6):421–34.
3. Main UEMS - Postgraduate Training. Cited 2022 Dec 15. Available from: https://www.uems.eu/areas-of-expertise/postgraduate-training.
4. EDAIC | ESAIC [Internet]. ESAIC; 2021. Cited 2022 Nov 26. Available from: https://www.esaic.org/education/edaic/
5. Jonker G, Manders LA, Marty AP, Kalkman CJ, ten Cate TJ, van Gessel EF, et al. Variations in assessment and certification in postgraduate anaesthesia training: a European survey. BJA Br J Anaesth. 2017;119(5):1009–14.
6. Yamamoto S, Tanaka P, Madsen MV, Macario A. Comparing Anesthesiology Residency Training Structure andRequirements in Seven Different Countries on Three Continents. Cureus. 9(2):e1060.
7. Martin JA, Regehr G, Reznick R, Macrae H, Murnaghan J, Hutchison C, et al. Objective structured assessment oftechnical skill (OSATS) for surgical residents. Br J Surg. 1997;84(2):273–8.
8. Zoller A, Hölle T, Wepler M, Radermacher P, Nussbaum BL. Development of a novel global rating scale for objectivestructured assessment of technical skills in an emergency medical simulation training. BMC Med Educ. 2021;21(1):184.
9. Fletcher G, Flin R, McGeorge P, Glavin R, Maran N, Patey R. Anaesthetists’ Non Technical Skills (ANTS): evaluation of a behavioural marker system†. BJA Br J Anaesth. 2003;90(5):580–8.
10. NASA Task Load Index | Digital Healthcare Research. Cited 2021 Nov 14. Available from: https://digital.ahrq.gov/health-it-tools-and-resources/evaluation-resources/workflow-assessment-health-it-toolkit/all-workflow-tools/nasa-.
11. Bhandary S, Lipps J, Winfield SR, Abdel-Rasoul M, Stoicea N, Pappada SM, et al. NASA Task Load Index scale toevaluate the cognitive workload during cardiac anesthesia based simulation scenarios. Int J Anesth Res. 2016;4(8):300–4.

**Table 1 (abstract O1).** See text for description

	**Germany (median (IQR))**	**Spain (median (IQR))**	***p*** **-value (Wilcoxon Rank-Sum Test)**
Knowledge test score	70.0 (10.0)	74.0 (11.0)	0.270
Clinical skills’ assessment score (scenario 1)	58.9 (10.0)	56.7 (8.9)	0.573
Clinical skills’ assessment score (scenario 2)	48.9 (14.4)	56.7 (13.3)	0.321
Non-technical skills’ assessment score (scenario 1)	3.4 (0.8)	3.3 (0.5)	0.705
Non-technical skills’ assessment score (scenario 2)	3.3 (0.6)	3.5 (0.7)	0.309
	**3** ^**rd**^ **year residents (median (IQR))**	**4th year residents (median (IQR))**	***p*** **-value (Wilcoxon Rank-Sum Test)**
Knowledge test score	70.0 (9.0)	78.0 (13.0)	**0.005**
Clinical skills’ assessment score (scenario 1)	53.3 (11.1)	59.4 (7.2)	**0.013**
Clinical skills’ assessment score (scenario 2)	47.8 (15.6)	56.7 (6.7)	**0.014**
Non-technical skills’ assessment score (scenario 1)	3.1 (1.0)	3.5 (0.5)	**0.012**
Non-technical skills’ assessment score (scenario 2)	3.2 (0.9)	3.5 (0.5)	**0.023**

## O2. Assessing Student Empathy Elicited After In-Person and Screen-Based Poverty Simulations

### Ashleigh Allgood^1^, Anita Samaniego-Avellana^1^, Dawn Taylor Peterson^1^, Michelle R. Brown^1^

#### ^1^University of Alabama at Birmingham

*Advances in Simulation 2024*, **9(1):**O2

***Introduction***

The University of Alabama at Birmingham championed a multidisciplinary pedagogical venture, the Poverty Simulation, to immerse students across various disciplines in realistic socio-economic scenarios. Over eight years, this initiative has engaged thousands of students to foster a deeper understanding of the barriers their future patients might encounter. Although the primary focus was on enhancing awareness of poverty-related barriers, we performed a retrospective examination of students' reflective evaluations post-simulation and classified their qualitative responses into a tripartite empathy framework.

***Description***

At our institution, we implemented a poverty simulation both in person and screen-based. Both modalities are followed by a structured debrief which focused on three objectives: 1) challenges faced navigating life in poverty, 2) decisions made that impacted family and income, 3) Insights gained as to how healthcare professionals can work together to meet the needs of individuals with low income. The debriefings are designed to promote interprofessional reflection and dialogue among students from diverse fields, enhancing the collaborative learning experience. Post-simulation, students are prompted to complete qualitative evaluations sharing lessons learned. These evaluations were retrospectively analyzed with empathetic statement classified into cognitive, emotional, or behavioral empathy.

***Results & Discussion***

It is important to train future healthcare providers how to empathetically care for those in socioeconomical hardship. In addition to confirming the simulations evoked empathy, further analysis was conducted to understand what type of empathy was elicited. We used the following definitions for our empathy framework:

- Cognitive: understanding what others think.
- Emotional: feeling what others feel.
- Behavioral: taking action to help others.

We had a three-person team classify qualitative data into the three types of empathy. Two people were involved in the original classification and a third person adjudicated any discrepancies. The analysis demonstrated that students revealed cognitive, emotional, and behavioral empathy during the simulation, irrespective of format. Overwhelmingly, students responded with cognitively empathic statements for both the in person and screen-based poverty simulations. For instance, one student noted ‘I gained insight as to how/why parents aren't able to always be active in their kid’s life,’ another remarked ‘Even if someone is doing their best, circumstance may still stand in the way of them doing what I think is right or best for them.’ Additional analysis will be shared in the presentation delving into a comprehensive review of the types of empathy evoked after participation in both modalities of the simulation.

***Keywords***

Empathy, Qualitative, Reflection

***Reference***

1. Empatico. Empathy Framework.2023; Available from: https://empatico.org/our-work/empathy-framework/

## O3. Assessment of Attitudes Towards Teamwork among Pharmacy Students in a COVID-19 Emergency Scenario: Interprofessional Virtual Simulation Training

### Krttin Bunditanukul^1^, Jiraphan Ritsamdang^1^, Navaporn Worashilchai^2^, Khuansiri Narajeenron^3^

#### ^1^Faculty of Pharmaceutical Sciences, Chulalongkorn, ^2^Faculty of Allied Health Sciences Chulalongkorn University, ^3^Faculty of Medicine, Chulalongkorn University

*Advances in Simulation 2024*, **9(1):**O3

***Introduction***

Effective teamwork is pivotal in emergencies, especially during unprecedented crises like the COVID-19 pandemic. Virtual simulation training presents an innovative approach to hone teamwork skills among pharmacy students preparing to practice in emergency departments. This study aimed to evaluate the impact of the simulation on improving teamwork attitudes among pharmacy students in a simulated severe COVID-19 pneumonia scenario within an emergency department.

***Methods***

Eleven pharmacy students in their third and fourth professional years participated in a COVID-19 emergency scenario using a computer-based 3D platform. This training encompassed introductory, pre-training, simulation, and debrief sessions spanning multiple days. Only one pharmacy student and five other healthcare students participated in each round, including one medical student, two nursing students, one medicinal technology and one radiology student. They managed a simulated patient with severe COVID-19 pneumonia in an emergency department. We conducted baseline and post-training assessments using the validated TeamSTEPPS Teamwork Attitudes Questionnaire (T-TAQ).

***Results & Discussion***

The overall mean baseline T-TAQ score was 120.45 ± 9.89, with significantly improved post-training at 132.37 ± 7.67 (Hedges' g = 1.506, *p* < 0.001). Specifically, three domains demonstrated statistically significant gains post-training. These included 'Team Structure,' which saw the most substantial improvement (Hedges' g = 2.435, *p* < 0.001), followed by ‘Mutual Support’ (Hedges' g = 1.397, *p* < 0.001), and ‘Situation Monitoring’ (Hedges' g = 0.765, *p* < 0.025), respectively. In conclusion, the COVID-19 pneumonia simulation training effectively enhances teamwork attitudes among pharmacy students, preparing them for collaborative roles in high-pressure emergency healthcare settings. This simulation approach, especially noteworthy in 'Team Structure,' 'Mutual Support,' and 'Situation Monitoring,' showcases the potential of virtual training technology to improve interprofessional collaboration. Future research should explore its long-term effects on preparedness and adaptability, further advancing pharmacy education and emergency care outcomes.

***Keywords***

Simulation Training; Virtual Simulation; Pharmacy Education; TeamSTEPPS

***Acknowledgements***

Acknowledgements to the ERVIPE Study Group and the Second Century Fund, Chulalongkorn University.

## O4. Challenges and opportunities in the uptake of simulation in healthcare education in the developing world: a scoping review

### ^1^Faisal Ismai, ^1^Khairulnissa Ajani, ^2^Pammla Petrucka

#### ^1^Aga Khan University Hospital, ^2^University of Saskatchewan

*Advances in Simulation 2024*, **9(1):**O4

***Introduction***

Simulation is increasingly being adopted by healthcare educators throughout the developed world to train clinical skills. While there is literature on learning via simulation in healthcare in the developed world, more studies are required to investigate the factors influencing this in developing countries. As simulation in healthcare education is resource intensive, a detailed analysis of the barriers and facilitators is imperative to inform how low to middle-income countries (LMIC) can implement sustainable simulation-enabled learning environments.

***Methods***

We conducted a scoping review to determine the key factors that act as deterrents as well as encouragement to the uptake of simulation as a teaching methodology in healthcare education in developing countries. The MEDLINE (using keywords and MeSH in OVID), PubMed (via NCBI using MeSH), and CINAHL databases were searched between January 2000 and December 2021 for research articles published in peer-reviewed English language journals. The final analysis yielded 47 articles. Challenges and opportunities were divided into professional, academic, and resource-based factors and their individual sub-themes.

***Results & Discussion***

The main challenges reported were the lack of a contextual curriculum, content-heavy curricula, dearth of trained simulationists and cost of simulators. Performance anxiety was an important challenge reported by both trainers and trainees, due to lack of familiarity not only with the teaching methodology but also the simulators. Main opportunities were an interest in adopting simulation-based education from both trainers and trainees, and the opportunity to improve patient safety and quality of education imparted. Other important findings were that academic leadership need to show interest and urgency to adopt simulation in curricula. Facilitators need to be developed and be provided with protected time to become simulationists. Local manufacturers need to be sourced for simulators, and transfer of technology and expertise needs to be negotiated with vendors. Simulation needs to be looked at from the lens of not only education, but more importantly, of patient safety in LMIC.

Our study utilized a robust scoping review process to highlight key areas of priority influencing the uptake of simulation in healthcare education. The fact that the conventional curriculum utilizing the traditional method of teaching and training remains in place in LMIC`s suggests that healthcare educators have yet to realize the full potential of simulation-based education. We provide a comprehensive evaluation of the factors that influence the uptake of simulation in healthcare education that can aid developing contexts to transition into simulation-enabled environments.

***Keywords***

Healthcare, Simulation, challenges, opportunities, developing, scoping

***References***

1. Aebersold M. The history of simulation and its impact on the future. AACN Adv Crit Care. 2016;27(1):56-61.
2. Ayaz O, Ismail FW. Healthcare Simulation: A Key to the Future of Medical Education - A Review. Adv Med EducPract. 2022;13:301-308.  10.2147/AMEP.S353777. PMID: 35411198; PMCID: PMC8994530.
3. Bogár PZ, Tóth L, Rendeki S, Mátyus L, Németh N, Boros M, Nagy B, Nyitrai M, Maróti P. The present and the futureof medical simulation education in Hungary. Orv Hetil. 2020;161(26):1078-87.
4. Martinerie L, Rasoaherinomenjanahary F, Ronot M, Fournier P, Dousset B, Tesnière A, Mariette C, Gaujoux S,Gronnier C. Health care simulation in developing countries and low-resource situations. J Contin Educ Health Prof. 2018;38(3):205-12.
5. Salman H. Most significant barriers and proposed solutions for medical schools to facilitate simulation-basedundergraduate curriculum in OBGYN. Arch Gynecol Obstet. 2021;304(6):1383-1386. 10.1007/s00404-021-06133-4.

**Fig. 1 (abstract O4).**
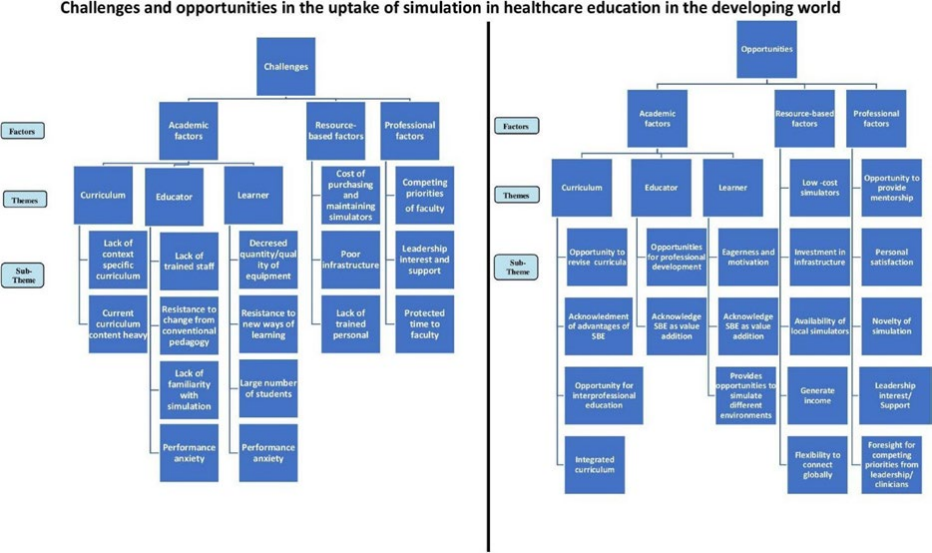
Challenges and opportunities in the uptake of simulation in healthcare education in the developing world

## O5. Comparison Of Hands On ORSIM Simulator Training Versus High Fidelity Mannequin Training Of Novice Anaesthesiology Residents For Fiberoptic Intubation Proficiency And Retention Ability: A Randomised Controlled Trial

### Nivedha Giridharan

#### All India Institute Of Medical Sciences

*Advances in Simulation 2024*, **9(1):**O5

***Introduction***

Fiberoptic bronchoscopy-guided intubation is crucial in managing difficult airways during anesthesia, enhancing safety and minimizing complications. However, limited training opportunities constrain anesthesiology residents' proficiency in FOB skills.

The primary aim is to compare intubation proficiency in normal airways using ORSIM Fiber Optic simulation and high-fidelity mannequin training, assessed by time to reach the carina. Secondary objectives included evaluating proficiency via the Global Rating Scale (GRS), assessing retention capacity and skills decay after 6 months, and measuring self-assessed confidence levels post-training.

***Methods***

This prospective randomised controlled study, conducted at AIIMS, New Delhi, involved 50 anaesthesia junior residents with over six months of training. They were randomly divided into two groups: the virtual reality simulator group (Group O, *n*=25) and the high-fidelity mannequin simulation group (Group H, *n*=25). Initial training encompassed airway anatomy and principles of flexible fiberoptic bronchoscope usage. Group O was trained on the ORSIM bronchoscopy simulator, while Group H used the AmbuScope on a high-fidelity mannequin. The primary measure of proficiency was the time taken to reach the carina during intubation, secondary assessments included assessing GRS for manipulation abilities. Retention capacity was evaluated at 1, 2, 4, and 6 months post-training. Participant confidence was assessed using Likert scores.

***Results & Discussion***

Time taken to reach the carina did not significantly differ between Group O and Group H immediately post-training (85.04 ± 29.72sec vs. 74.64 ± 53.03sec, p = 0.39). After 6 months, a significant difference emerged (58.34 ± 18.23sec vs. 43.70 ± 12.87sec, *p* = 0.001).GRS showed no significant difference between Group O and Group H immediately post-training (2.28 ± 0.61 vs. 2.28 ± 0.73, *p* = 1.00). Self-assessment scores immediately after the training session revealed a statistically significant difference between Group O and Group H with scores of (1.92 ± 0.27) and (2.56 ± 0.58) respectively (*p* = 0.01). Post 6 months of training, there was a significant difference in GRS between Group O and Group H (4.52 ± 0.50) vs (4.16 ± 0.37) respectively (*p* = 0.006). The study compared virtual reality (VR) and high-fidelity mannequin simulation for fiberoptic bronchoscopic-guided intubation training. Initially, no significant difference was observed in intubation time. However, after 6 months, participants in the VR group took longer to reach the carina, highlighting potential differences in skill retention. Global Rating Score (GRS) showed no significant variance, implying similar technical proficiency. Notably, participants in the VR group reported higher initial confidence, emphasising the psychological impact of the training modality. In conclusion, the study underscores the importance of long-term skill retention assessment and suggests a blended training approach for optimal outcomes.

***Keywords***

Fiberoptic bronchoscopy

***References***

1. Jiang B, Ju H, Zhao Y, Yao L, Feng Y. Comparison of the efficacy and efficiency of the use of virtual reality simulationwith high-fidelity mannequins for simulation-based training of fiberoptic bronchoscope manipulation. Simulation in Healthcare. 2018 Apr 1;13(2):83-7.
2. Baker PA, Weller JM, Baker MJ, Hounsell GL, Scott J, Gardiner PJ, Thompson JM. Evaluating the ORSIM® simulatorfor assessment of anaesthetists' skills in flexible bronchoscopy: aspects of validity and reliability. BJA: British Journal of Anaesthesia. 2016 Sep 1;117(suppl_1):i87-91.

**Table 1 (abstract O5).** Performance comparison between two groups

	Group O (Mean ± SD)	Group H (Mean ± SD)	*P* Value
Time to reach carina (seconds)	85.04 ± 29.72	74.64 ± 53.03	*p* = 0.39
Time to reach carina (6 Months Later)	58.34 ± 18.23	43.70 ± 12.87	*p* = 0.001
GRS	2.28 ± 0.61	2.28 ± 0.73	*p* = 1.00
GRS (6 Months Later)	3.8 ± 0.40	3.92 ± 0.27	*p* = 0.22
Self-Assessment Score	1.92 ± 0.27	2.56 ± 0.58	*p* = 0.01
Self-Assessment Score (6 Months Later)	4.52 ± 0.50	4.16 ± 0.37	*p* = 0.006

## O6. Comparison of laryngeal mask airway and endotracheal tube use in inhospital cardiopulmonary arrest due to cardiac reasons: Pediatric simulation-based randomized study

### Ahmet Kagan Özkaya

#### Karadeniz Technical University

*Advances in Simulation 2024*, **9(1):**O6

***Introduction***

Laryngeal mask airway (LMA) can be used safely in infants and children, however, there are a limited number of studies comparing LMA and endotracheal intubation in the resuscitation of pediatric patients that can provide safe recommendations. The aim of this study is to reveal the effects of the use of LMA on the resuscitation process and whether it shortens the effective ventilation and defibrillation time compared to the endotracheal tube in pediatric simulated inhospital cardiopulmonary arrest due to cardiac cause.

***Methods***

This study was designed as crossover randomized. Before the study, all participants received a full day of training on advanced child life support, including theorical and practical training. All participants completed two separate scenarios involving two different drug intoxications involving a high-fidelity pediatric simulation mannequin. The scenarios were essentially cases of cardiopulmonary arrest due to ventricular fibrillation, with the same management. At the end of one scenario, the next scenario was started without a break, and a debriefing session was held with the participant at the end of the scenarios. In one scenario, the participant used an endotracheal tube as an advanced airway device, and in the other, the LMA. Demographic, educational and medical experience characteristics of the participants their were recorded. During the simulation, the entire performances were recorded with video and audio recordings. Outcomes of the study are also were determined. A mini survey was administered to all participants, questioning their training status and experiences before the simulation, as well as revealing their anxieties before and after the simulation scenarios. In line with previous studies, sample size was calculated that at least 16 participants would take part in this study for alpha=0.05 and (1-beta)=0.80.

***Results & Discussion***

The study was conducted with 17 participants. The average age of the participants was 30.2±2.7 years and 8 (47.1%) were male and 9 (52.9%) were female. While the effective ventilation time using the endotracheal tube was 73.88±51.68 seconds, it was 38.29±35.21 seconds with LMA (*p*=0.001). Additionally, when the time until defibrillation was compared between the endotracheal tube and LMA groups, a statistically significant difference was observed between them (81.47±43.28 seconds; 48.70±35.88 seconds, respectively; *p*=0.04). Table 1 includes comparisons of study outcomes. In conclusion, the use of LMA may allow faster effective ventilation and early defibrillation compared to an endotracheal tube in simulated pediatric arrests. Therefore, the use of LMA may be a promising approach in cardiopulmonary pediatric arrests due to cardiac causes.

***Keywords***

endotracheal tube, laryngeal mask airway, pediatric arrest, simulation

***References***

1. Khalil PA, Berkovich J, Maniaci V, Lozano JM, Lowe DA. Performance of emergency medical service providers inpediatric and adult simulation of unstable supraventricular tachycardia. Pediatr Emerg Care. 2020;36(8):451–5.
2. Lee SY, Hong JY, Oh JH, Son SH. The superiority of the two-thumb over the two-finger technique for single-rescuerinfant cardiopulmonary resuscitation. Eur J Emerg Med. 2018;25(5):372–6.
3. Restrepo CG, Baker MD, Pruitt CM, Gullett JP, Pigott DC. Ability of pediatric emergency medicine physicians toidentify anatomic landmarks with the assistance of ultrasound prior to lumbar puncture in a simulated obese model. Pediatr Emerg Care. 2015;31(1):15–9.
4. Sudikoff SN, Overly FL, Shapiro MJ. High-fidelity medical simulation as a technique to improve pediatric residents’emergency airway management and teamwork: A pilot study. Pediatr Emerg Care. 2009;25(10):651–6.

**Table 1 (abstract O6).** Comparisons of study outcomes according to advanced airway tools

Parameters	Endotracheal Entubation	Laryngeal Mask Airway	*p*
First effective ventilation begining time with advanced airway tool, mean SD, s	73.88 ± 51.68	38.29 ± 35.21	**0.001**
Time to connect to defibrillator, mean SD, s	11.41± 3.02	12.64 ± 4.06	0.56
Time to antiarrhythmic drug request, mean SD, s	8.82 ± 36.38	16 ± 54.41	0.46
Time to rhythm identification, mean SD, s	27.05 ± 46.28	15.52 ± 8.49	0.56
Time to identification unstable patient, mean SD, s	14.52 ± 9.32	11.82 ± 5.06	0.48
Time to start CPR, mean SD, s	26.11 ± 16.12	20.29 ± 9.35	0.16
Time until defibrillation begins, mean SD, s	81.47 ± 43.28	48.70 ± 35.88	**0.04**
First adrenaline administration time, mean SD, s	73.41 ± 51.21	57.70 ± 35.76	0.27
Number of multiple advanced airway attempts, n (%)	2 (11.7)	-	-
Antiarrhythmic drug request, n (%)	1 (5.9)	3 (17.6)	-
Electrical intervention, n (%)			
Synchronized	1 (5.9)	1 (5.9)	-
Unsynchronized	16 (94.1)	16 (94.1)	-
Is the defibrillation dose adequate?			
Yes	14 (%82.4)	12 (%70.6)	-
No	3 (%17.6)	5 (%29.4)	
Was appropriate defibrillation performed? (safety precaution-warnings)			
Yes	17 (%100)	17 (%100)	-
No	-	-	
Anxiety level before scenarios, median (IQR)	7 (3-8)	6 (3-8)	0.85
Anxiety level after scenarios, median (IQR)	7 (3-9)	6 (3-8)	0.40

*SD* Standart deviation, *CPR* Cardiopulmonary resusitation, *IQR* Interquartile range

Statistical significance was considered when *p <* 0.05

## O7. Crossing boundaries in the chain of child safeguarding- interprofessional simulation using design-based research

### Michelle O'Toole^,^ Walter Eppich, Clare Sullivan, Naoise Collins, Claire Mullhall, Dani Hall, Aideen Walsh, Michelle Whelan, Andrea Doyle

#### RCSI University of Medicine and Health Sciences

*Advances in Simulation 2024*, **9(1):**O7

***Introduction***

Worldwide, over 1 billion children/year experience violence and abuse, causing long-term emotional, social, and economic consequences, including over 40,000 deaths/year. Even after children at-risk for abuse are identified, the system meant to protect them fails them, mostly due to communication breakdowns between professionals, including those from health/social care as well as first responders. This problem impacts health professionals in emergency departments (ED) who commonly evaluate non-accidental injuries and non-specific signs and symptoms of child abuse and must make appropriate referrals to ensure ongoing evaluation of legitimate concerns to prevent further harm and death.

This study explores learning across professional boundaries between doctors, nurses and social workers to explore their own and others’ roles and valuable contributions to child safeguarding in emergency department settings to promote interprofessional and cross-disciplinary teamwork and communication.

The course had two aims: (a) to prepare emergency professionals to contribute to the care of abused children through effective interprofessional teamwork and collaboration, and (b) to advance the science of child safeguarding education by using DBR

***Methods***

Using sociocultural learning theory and design-based research, we designed, implemented, and evaluated three iterations of a 1.5-day simulation-based interprofessional child safeguarding course. Key stakeholders co-designed the course and served as co-faculty. We collected:

ethnographic observations; semi-structured interviews (participants, faculty and simulated parents); quantitative questionnaires (psychological safety and interprofessional collaboration).

We used reflexive/theoretical thematic analysis to analyse qualitative data from interviews supplemented by and also guided by field observations.

After each iteration of the course, we analysed and synthesized quantitative and qualitative data to make informed decisions about course modification. Given the low sample size, our rich qualitative data predominantly guided design choices.

***Results & Discussion***

32 participants completed the course. Key themes included:

1. Interprofessional collaboration enabled learning about respective roles and responsibilities;
2. Psychological safety promoted boundary crossing;
3. Use of precise language and approaches to interprofessional communication fostered learning;

Transfer of learnings to the workplace required expansive thinking, strategic silence, and better questions. Using DBR, we demonstrated the importance of interprofessional training in child safeguarding and the benefits of bringing together physicians, nurses and social workers to enhance collaboration and effective information sharing. This innovative course has broad applicability to all professionals working with children; frontline/emergency services, legal professionals and teachers.

***Keywords***

interprofessional simulation; nursing; social work; emergency medicine

***References***

1. Ainsworth, F., & Hansen, P. (2011). The Munro Review of Child Protection: Final Report — A Child-Centred System: A Review and Commentary. In Children Australia (Vol. 36, Issue 3). 10.1375/jcas.36.3.164.
2. Crawford, A., & L’Hoiry, X. (2017). Boundary crossing: networked policing and emergent ‘communities of practice’ in safeguarding children. Policing and Society, 27(6), 636–654.
3. De Nooijer, J., Dolmans, D. H. J. M., & Stalmeijer, R. E. (2021). Applying Landscapes of Practice Principles to the Design of Interprofessional Education. Teaching and Learning in Medicine, 0(0), 1–6. 10.1080/10401334.2021.1904937.
4. Green, P. (2019). The role of designated and named professionals in child safeguarding. Paediatrics and Child Health (United Kingdom), 29(1), 1–5. 10.1016/j.paed.2018.11.008.
5. Hood, R., Price, J., Sartori, D., Maisey, D., Johnson, J., & Clark, Z. (2017). Collaborating across the threshold: The development of interprofessional expertise in child safeguarding. Journal of Interprofessional Care, 31(6), 705–713. 10.1080/13561820.2017.1329199.
6. HSE 2019 HSE Child Protection and Welfare Policy https://www.hse.ie/eng/services/list/2/primarycare/childrenfirst/resources/hsecpwpolicy.pdf https://www.tcd.ie/tricc/assets/pdfs/crc-archive/2006-Buckley-Horwath-Whelan-Framework-Assessment-Vulnerable.pdf

## O8. Current state of Peruvian simulation centers: ASPEFAM national survey results

### Victor Andrés Velásquez Rimachi, Alvaro Prialé Zevallos, Solange Dubreuil Wakeham, Daniela Samaniego Lara

#### Universidad Científica del Sur^1^

*Advances in Simulation 2024*, **9(1):**O8

***Introduction***

Latin American medical curricula have increasingly integrated simulation-based education (1). In Peru, however, its adoption varies. While some institutions have fully embraced it, its broader influence remains limited. To address this, the "Peruvian Association of Medical Faculties" (ASPEFAM) set up a clinical simulation center network in 2017 (2). This initiative seeks to elevate health professional training through simulation. Our study examines the status of these centers in Peru, focusing on resources, teaching approaches, and ongoing medical education. We delve into directors' views on their centers strengths and priorities.

***Methods***

We conducted a cross-sectional survey using a digital questionnaire in Spanish, aimed at the 34 directors of

ASPEFAM-affiliated Peruvian simulation centers. Expert simulation professionals curated the survey content, rooted in established parameters for detailing simulation resources and operations.

***Results & Discussion***

Out of 34 faculties, 33 responded, encompassing 17 private and 16 public institutions. 51% have practiced simulation for over five years, with 95% having designated simulation spaces. Every institution integrates simulation in undergraduate studies; 19% extend it to postgraduate courses. Thirty-two faculties conduct a face-to-face simulation, with 14 using standardized patient simulation. Eleven faculties use distinct applications of virtual patient software and augmented reality to practice virtual simulation. Ten faculties use hybrid simulation, and seven faculties use telesimulation.

Regarding health careers utilizing simulation, medicine is predominant in 32 faculties, trailed by nursing in 15. Only 26% of faculties have >5 full-time teaching staff. Evaluation techniques vary; ECOE is the primary method for 18 faculties. 72% reported that only between 1-25% of their staff have received prior training in simulation. The most common types of simulators used are task trainers and low-fidelity full-body simulators. Task trainers and basic full-body simulators are the most used tools. Directors frequently cited strengths in “equipment”, “infrastructure”, and “skill simulation”. However, the most significant need identified was teacher training, highlighted by 25 faculties.

In summary, Peru's simulation centers offer diverse simulation activities, predominantly for undergrad medical students.

Their potential is somewhat limited by insufficient staff training that doesn't meet Peru's educational requirements (3). The prevailing need for continuous training could be addressed by fostering national and international collaborative networks. We must continue with the accreditation processes in Peru with objective monitoring of compliance with quality standards.

***Keywords***

Simulation Training; Developing Countries; Perú

***References***

1. Armijo-Rivera S, Machuca-Contreras F, Raul N, de Oliveira SN, Mendoza IB, Miyasato HS, Díaz-Guio DA.
2. Characterization of simulation centers and programs in Latin America according to the ASPIRE and SSH quality criteria. Advances in Simulation. 2021 Dec;6(1):1-1.
3. ASPEFAM.Red Nacional de Centros de Simulación Clínica. (n.d.). Retrieved October 28, 2023, fromhttps://www.aspefam.org.pe/redncsc/
4. Chernikova O, Heitzmann N, Stadler M, Holzberger D, Seidel T, Fischer F. Simulation-based learning in highereducation: A meta-analysis. Review of Educational Research. 2020 Aug;90(4):499-541.

## O9. Defragmented debriefing Methodology for Enhancing Online Simulation Training in Medical Education

### Natalia Lopina

#### Director of the Simulation Training Platform for Medical Education “ClinCaseQuest”

*Advances in Simulation 2024*, **9(1):**O9

***Introduction***

Medical education has witnessed a paradigm shift towards online learning, necessitating innovative approaches to bridge the gap between theoretical knowledge and clinical practice. This abstract presents an innovative staged debriefing methodology designed to enhance the efficacy of online simulation training in medical education. The primary aim of this methodology is to simulate real clinical decision-making processes within an online environment, fostering critical thinking, problem-solving skills, and clinical competence among medical learners.

***Description***

The staged debriefing methodology integrates seamlessly with online simulation scenarios, helping to create an adaptive learning strategy.

We developed the methodology for staged debriefing which means demonstration of the consequences of the decisions made, emotional involvement, reasoning, explanations, and feedback inside the training scenario. Our approach is based on two strategies of staged debriefing according to the patient’s care or the patient’s way in particular nosology. The methodology of staged debriefing encourages learners to critically analyze their choices and the consequences therein. This method effectively emulates real-time clinical decision-making by recreating scenarios with escalating challenges.

***Results & Discussion***

Preliminary implementation of the staged debriefing methodology demonstrates promising outcomes. Learners reported heightened engagement and an improved understanding of the clinical reasoning process. The adaptive nature of the methodology allowed learners to delve into varying complexities based on their proficiency, fostering a personalized learning experience. Initial assessment also revealed a positive impact on learners' confidence and decision-making skills, crucial for effective clinical practice. The innovative staged debriefing methodology represents a significant advancement in online simulation training for medical education. By simulating real clinical decision-making processes, it bridges the gap between theoretical learning and practical application. This methodology offers educators a powerful tool to cultivate critical thinking, clinical competence, and problem-solving skills in medical learners. As medical education continues to evolve, the staged debriefing methodology stands as a pivotal approach to enhance online simulation training, ultimately contributing to the development of proficient and confident healthcare professionals.

***Keywords***

defragmented debriefing, simulation-based education, simultaneous debriefing, online clinical simulation, in-simulation debriefing, competency formation, medical errors, competency-based learning, clinical simulation, clinical case learning, problem-based learning, simulation-based learning, scenario-based learning, clinical reasoning, clinical thinking, decision-making, adaptive learning; internal medicine; emergency care cardiology; neurology; haematology.

***Reference***

1. Lopina N. “Staged Debriefing in Simulation Training in Medical Education for Competencies Formation” / 28th Annual conference Society for Simulation in Europe SESAM 2023/ Lisbon, Portugal, 14-16 June 2023

## O10. Domestic Violence simulation for Scottish General Practice Teams

### Tiffany Keep, Sarah Luty

#### NHS Education for Scotland Medical Education Fellow,

*Advances in Simulation 2024*, **9(1):**O10

***Introduction***

Whilst a criminal offence in the UK, cases of domestic violence continue to rise (1, 2) and patients experiencing domestic violence present to their GP. Previous work by the authors showed lack of preparedness in GP trainees and the value of simulation based learning to improve skills in managing presentations of domestic violence.

This study aimed to develop and evaluate a simulation programme to help GP teams- reception staff, healthcare support workers, managers, nurses and General practitioners recognise and manage cases involving domestic violence.

***Description***

In situ simulation scenarios were designed around three core domestic violence presentations, using specialist actors to enable the scenarios. Three scenarios included one participant, with one scenario as a group case. Simulations lasted 10-15 minutes, and were followed by a debriefing by an experienced GP facilitator.

Scenario 1 was based on coercive and control; where a partner is witnessed by reception staff exhibiting behaviours that intimidate, manipulate and threaten the patient before her scheduled appointment.

Scenario 2 was physical violence with bruising noted by the healthcare support worker or receptionist during a routine blood pressure check for the contraceptive pill.

Scenario 3 was based on a potential grooming, where staff are given information about young adolescent girls in vulnerable situations.

Written feedback was collected from participants to drive improvement of the simulation experience.

The intended learning outcomes were based on the Scottish adult support and protection framework for all health and social care staff in Scotland.

***Results & Discussion***

The initial pilot was successful with the practice feedback strongly agreed that the session will help their future care of vulnerable groups. Free text comments included “interesting and realistic scenarios” and safe learning environment for challenging topic” Increased preparedness of what to say “in the now” .GP teams valued the opportunity to learn together and share experience.

The main learning points were 1. highlighting role of reception and administrative staff in observing behaviours not witnessed by clinical staff. 2. Increased awareness of duty to report concerns . 3. lack of pathways and protocols for information sharing between practice team members. 4. lack of recording and coding of domestic violence or vulnerable adults.

Further pilot practices have been recruited, but barriers to scaling up have been encountered with difficulty in accessing protected time within general practice, lack of perceived value of including wider team in simulation based learning events, and a lack of awareness of legislative framework for protection of vulnerable adults.

***Keywords***

Primary care, vulnerable groups

***References***

1. Statistics OfN. Domestic abuse prevalence and trends, England and Wales: year ending March 2021: Office forNational Statistics; 2021 [Long-term trends and types of domestic abuse experienced by adults, based on findings from police recorded crime.]. Available from: Domestic abuse prevalence and trends, England and Wales Office for National Statistics (ons.gov.uk).
2. Government S. Domestic abuse: statistics recorded by the Police in Scotland - 2020/21: Scottish Government; 2021 [Available from: Key points - Domestic abuse: statistics recorded by the Police in Scotland - 2020/21 - gov.scot (www.gov.scot).

## O11. Evaluation of cardiac and muscular parameters during chest compressions: an international multicentric pseudo-randomised manikin trial

### Ingrid Bispo^1^, Abel Nicolau^1^, Marc Lazarovici^2^, Christoffer Ericsson^3^, Max Gottstein^2^, Frieder Pankratz^2^, Pedro Vieira-Marques^1^, Carla Sá-Couto^1^

#### ^1^Faculty of Medicine, University of Porto (FMUP), LMU Universiry Hospital^2^, University of Helsinki, Faculty of Medicine^3^

*Advances in Simulation 2024*, **9(1):**O11

***Introduction***

Cardiopulmonary resuscitation (CPR) demands a moderate physical effort from the rescuer and induces physiological and muscular body adaptations. Previous studies [1-3] showed that heart rate variability and muscle fatigue are related to a decrease in CPR quality, and this varies regarding the rescuer’s position. The aim of this study is to evaluate the real-time changes in cardiac and muscular parameters during chest compressions (CC) in 4 different rescuer’s position and to examine whether they differ among the groups.

***Methods***

This international multicentric pseudo-randomised manikin study was conducted in Portugal, Finland and Germany, between May and October 2023. Healthcare professionals experienced in CPR aged between 18 and 65 years old were recruited by convenience sampling to perform uninterrupted 3 minutes of chest compressions in a manikin (Laerdal Resusci Anne QCPR). Subjects reporting back pain and pregnant women were excluded. Participants were pseudo-randomized into 4 different group settings: (1) manikin laying on the ground and rescuer with knees on the floor; (2) manikin laying on a lowered bed and rescuer with no step stool; (3) manikin laying on a bed and rescuer with step stool; and (4) manikin laying on a bed and rescuer with knees on the bed. In standing positions, the height of the bed was adjusted to the participant’s knees. No mattress was used to prevent CC damping. Sociodemographic data were collected using a questionnaire. Electrocardiogram (ECG) electrodes were used to measure real-time heart rate (HR) and the intervals between RR peaks (RRi), electromyogram (EMG) electrodes were placed in both triceps brachii to collect the maximum voluntary contraction (MVC) and the root mean square (RMS) from the muscle action potentials.

Approval from the ethical committee was obtained prior to the study.

***Results & Discussion***

Sixty-two subjects were included with different backgrounds (nurses, medical doctors and paramedics). Mean age was 35 years old (± 8,6) and mean BMI was 25 Kg/m2 (± 4,5). Most of participants were female (53,2%). Figure 1 shows the changes in cardiac and muscular parameters. Heart rate increased during the CPR, and RRi declined proportionally.

MVC and RMS showed minimal variability over time. No significant differences were found in any variable among the 4 groups. Conclusion: This study shows that changes in cardiac and muscular parameters during CPR are similar among different rescuer’s position. Additional tests, examining the correlation between CPR quality measures and rescuer fatigue, while considering factors such as gender, profession, and country, are currently in progress.

***Keywords***

cardiopulmonary resuscitation; chest compressions; muscular contraction; muscular fatigue, heart rate variability.

***Acknowledgements***

This work was supported by national funds of the FCT – Fundação para a Ciência e a Tecnologia, I.P., under the project “QualityCPR, ref. 2022.03731.PTDC”, and by a grant from the Laerdal Foundation (ref. 2022-0083).

***References***

1. Riera SQ, González BS, Álvarez JT, Fernández MF and Saura JM. The physiological effect on rescuers of doing 2 min of uninterrupted chest compressions. Resuscitation. 2007;74:108 – 112.
2. Bae GE, Choi A, Beom JH, Kim MJ, Chung HS, Min IK et al. Correlation between real-time heart rate and fatigue inchest compression providers during cardiopulmonary resuscitation. Medicine. 2021;100(16): (e25425).
3. Cho Y, Lee, Y, Lim TH, Chee Y, Oh J, Kim W et al. What muscles need to be trained for high-quality chestcompression? Australasian Emergency Care. 2020: 272 – 280.

**Fig. 1 (abstract O11).**
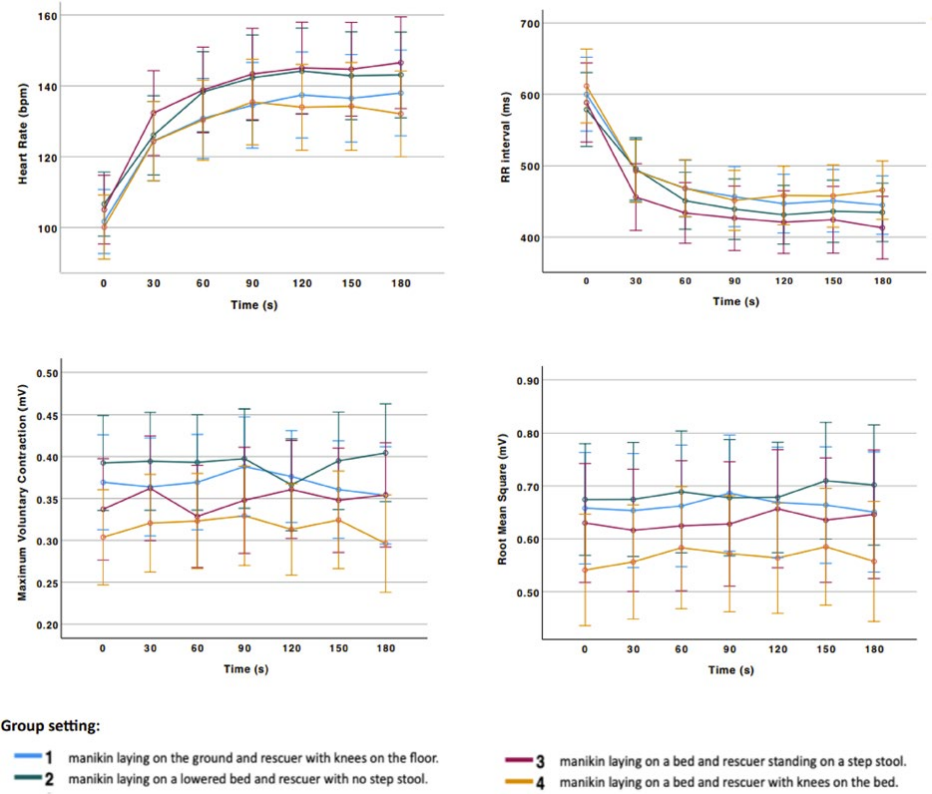
Changes in cardiac and muscular parameters over 3 minutes of chest compressions, in 4 different rescuer’s position

## O12. Exploring self-led debriefings in simulation-based education: An integrative review

### Prashant Kumar^1^, Susam Somerville^2^

#### University of Dundee, NHS Greater Glasgow & Clyde^1^, University of Dundee^2^

*Advances in Simulation 2024*, **9(1):**O12

***Previous publication*** - Exploring in-person self-led debriefings for groups of learners in simulation-based education: an integrative review | Advances in Simulation | Full Text (biomedcentral.com)

## O13. Exploring the influence of rescuer position on chest compression quality: an international multicentric pseudo-randomised manikin trial

### Abel Nicolau^1^, Ingrid Bispo^2^, Marc Lazarovici^3^, Christoffer Ericsson^4^, Jennifer Tempfli^3^, Selina Kim^3^, Inês Jorge^1^, Pedro Vieira-Marques^1^, Carla Sa-Couto^1^

#### ^1^CINTESIS@RISE, Faculty of Medicine, University of Porto, Porto, Portugal, ^2^Community Medicine, Information and Decision Sciences Department (MEDCIDS), Faculty of Medicine, University of Porto (FMUP), Porto, Portugal, ^3^institute for Emergency Medicine and Management in Medicine – INM, LMU University Hospital, Munich, Germany, ^4^Arcada University of Applied Sciences, School of Business and Healthcare, Helsinki, Finland; University of Helsinki, Faculty of Medicine, Helsinki, Finland

*Advances in Simulation 2024*, **9(1):**O13

***Introduction***

The key to successful resuscitation lies on the prompt onset of the life-saving maneuvers combined with their quality and effectiveness[1]. The position of the rescuer and the victim’s placement influences the quality of compressions[2]. The rescuer needs to assume a specific position based on the victim’s placement, which can impair the needed strength to achieve the recommended compressions depth or result in excessive pressure hindering full chest recoil. The aim of this study is to explore the influence of different positions of the rescuer in CPR quality, over time.

***Methods***

This international multicentric pseudo-randomised manikin trial was carried-out between May and October 2023, in Portugal, Germany and Finland. Participants were recruited by convenience sampling and pseudo-randomized into 4 independent groups, based on a specific setting: (1) manikin laying on the ground and rescuer with knees on the floor; (2) manikin laying on a lowered bed and rescuer standing on the floor; (3) manikin laying on a lifted bed and rescuer standing on a step stool; and (4) manikin laying on a bed and rescuer with knees on the bed. The height of the bed was adjusted to the selected setting, aligning it with the rescuer’s knee level. No mattress was used. Each participant performed 3 minutes of uninterrupted chest compressions in a manikin (Laerdal Resusci Anne QCPR). Demographic details were collected using a questionnaire. The inclusion criteria were healthcare professionals, aged between 18 and 65 years old, self-reporting good general health and physical condition, and experience in CPR. Pregnant women and participants reporting fatigue/muscle pain were excluded. Approval from the ethical committee was obtained prior to the study.

***Results & Discussion***

Fifty-one healthcare professionals, including medical doctors, nurses and paramedics, participated in this study. Their mean age was 34.08±8.39 years, with 24 of them being female. Figure 1 presents the variation of CPR parameters (depth, recoil, frequency and overall score) over time, in different positions. No statistically significant difference was found between groups. Overall, most of the participants adhered to the current guidelines, demonstrating adequate performance in all CPR parameters, regardless of position. The compression depth mean shows a consistent decrease in all groups, with a concomitant increase in the standard deviation, indicating a greater performance variation over time. This study suggests that performance is not influenced by the position of the rescuer. Further analysis of the results, considering gender, profession, and country, is currently being undertaken.

***Keywords***

emergency medicine, cardiopulmonary resuscitation, quality, chest compressions

***Acknowledgements***

This work was supported by national funds of the FCT – Fundação para a Ciência e a Tecnologia, I.P., under the project “QualityCPR, ref. 2022.03731.PTDC”, and by a grant from the Laerdal Foundation (ref. 2022-0083).

***References***

1. Olasveengen, T. M., Semeraro, F., Ristagno, G., Castren, M., Handley, A., Kuzovlev, A., ... & Perkins, G. D. (2021). European resuscitation council guidelines 2021: basic life support. Resuscitation, 161, 98-114.
2. Mygind-Klausen, T., Jæger, A., Hansen, C., Aagaard, R., Krogh, L. Q., Nebsbjerg, M. A., ...& Løfgren, B. (2018). In abed or on the floor?–The effect of realistic hospital resuscitation training: A randomised controlled trial. The American journal of emergency medicine, 36(7), 1236-1241.

**Fig. 1 (abstract O13).**
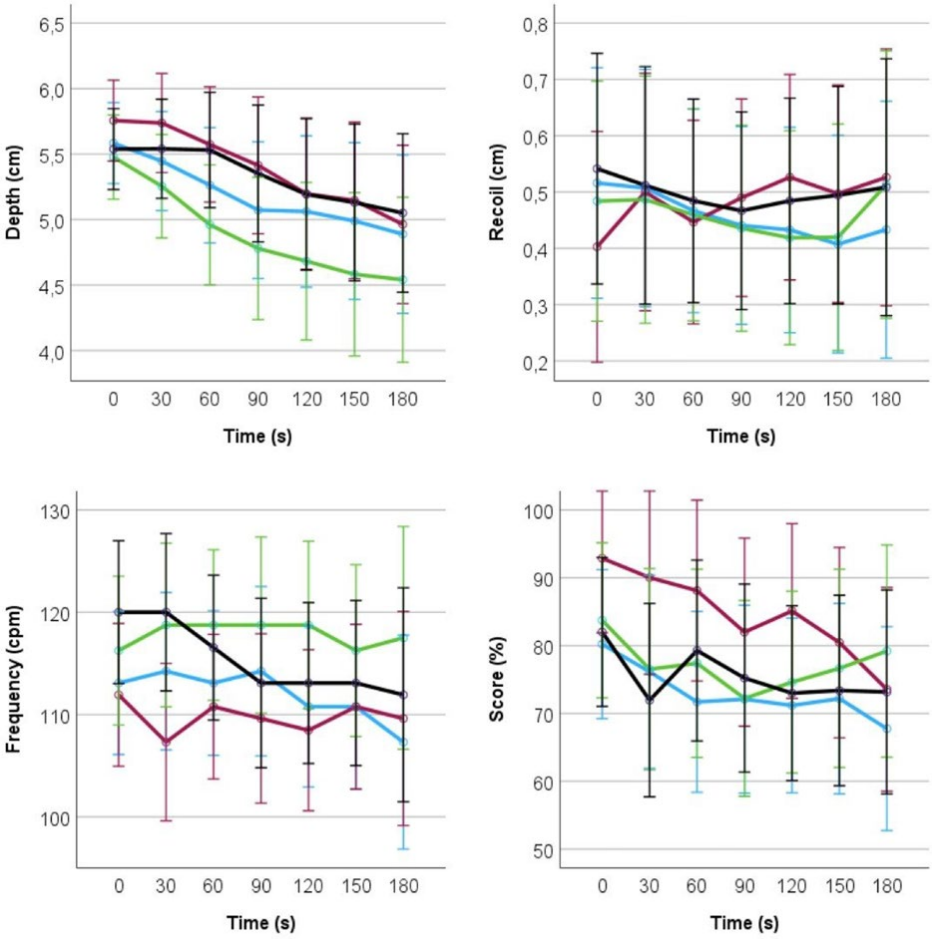
Over time variation of CPR parameters (depth, recoil, frequency and score) in different positions. Blue – manikin laying on the ground and rescuer with kneers on the floor; Red – manikin laying on a lowered bed and rescuer with no step stool; Green – manikin laying on a bed and rescuer standing on a step stool; Black – manikin laying on a bed and rescuer with knees on the bed

## O14. Faculty Development for Translational Simulation: a qualitative study of current practice

### Victoria Brazil^1^, Eve Purdy^1^, Alexander El Kheir^2^, Rebecca Szabo^3^

#### ^1^Bond University, ^2^Gold Coast Health, ^3^University of Melbourne

*Advances in Simulation 2024*, **9(1):**O14

**Previous publication-** https://advancesinsimulation.biomedcentral.com/articles/10.1186/s41077-023-00265-0

## O15. Human factor skills - From Simulation-based training to competency in clinical practice

### Lotte Abildgren

#### Odense University Hospital, Denmark; University of Southern Denmark; Hospital Sønderjylland

*Advances in Simulation 2024*, **9(1):**O15

***Introduction***

Simulation-based training has become increasingly prevalent as a tool to augment pregraduate healthcare students' knowledge and human factor skills. While its efficacy in imparting technical skills to established qualified healthcare personnel is acknowledged, there remains a gap in understanding transfer of the learned human factor skills to competency in clinical practice. This study aimed to shed light on how qualified in-hospital healthcare personnel integrate skills from simulation-based training into their clinical roles.

***Methods***

The study used a qualitative, phenomenological-hermeneutical approach to explore transfer of newly learned human factor skills. The study spanned three phases of ethnographic examination: clinical, simulation-based training, and transfer. Each phase was built upon insights from incipient analysis of the prior phase to understand the transfer process. The research utilised a comprehensive dataset of roughly 107 hours of video recordings, field notes, and in-depth reflective dialogues among the research team. The data analysis was conducted by a hybrid method of two qualitative methodologies: Ricœur-Inspired Analysis and Cognitive Event Analysis.

***Results & Discussion***

The research emerged three central themes regarding transfer of human factor skills:

Individual Transfer of Learning Intercollegiate Transfer of Learning

Organisational Transfer of Learning.

While individuals and intercollegiate groups displayed varying degrees of skill transfer, a foundational finding was the indispensable role of organisational structures. Transferring human factor skills from learning to clinical competency requires an organisational framework to scaffold the process. The study identified a notable gap in this area: the absence of organisational emphasis on human factor skills transfer resulted in a lack of motivation and effort to transfer the newly acquired skills into competency.

The findings provide a more profound comprehension of qualified healthcare personnel's hurdles in transferring human factor skills from a simulated environment to clinical contexts. The research emphasises the essential importance of organisations in this process. For the potential of simulation-based training to be realised fully, healthcare institutions need to recognise the weight of human factor skills, emphasise a team-based approach to the transfer process, and provide the necessary infrastructural support. This will facilitate a seamless transfer of skills and support patient care's quality and safety standards.

***Keywords***

Human factor skills; transfer; ethnography; qualified healthcare personnel; competency

***References***

1. Abildgren L, Lebahn-Hadidi M, Mogensen CB, Toft P, Nielsen AB, Frandsen TF, et al. The effectiveness of improving healthcare teams’ human factor skills using simulation-based training: A systematic review. Advances in Simulation. 2022;7(1):12.
2. Abildgren L, Lebahn-Hadidi M, Mogensen CB, Toft P, Steffensen SV, Hounsgaard L. Transfer of human factor skills from simulation-based training to competency in clinical practice–a demonstration of a hybrid method for assessing transfer of learning. International Journal of Healthcare Simulation. 2023(null):1-13.
3. Lebahn-Hadidi M, Abildgren L, Hounsgaard L, Steffensen SV. Integrating cognitive ethnography and phenomenology: rethinking the study of patient safety in healthcare organisations. Phenomenology and the Cognitive Sciences. 2021;22(1):193-215.

## O16. Implementing a psychiatry simulation course in rural Scotland

### Neera Gajree, Kenneth Ruddock

#### NHS Lanarkshire

*Advances in Simulation 2024*, **9(1):**O16

***Introduction***

Nearly one million people live in rural areas in Scotland (1). With vulnerability to loneliness, poor accessibility to high quality support and stigma acting as a barrier to seeking care in close knit communities, mental health is a recurring issue in rural Scotland. It has also been noted that many healthcare professionals in remote areas do not have mental health training (2).

Since 2009, NHS Education for Scotland has operated the Mobile Skills Unit (MSU), a training unit which brings state-of-the-art immersive simulation facilities to rural Scotland (3). In order to support staff working rurally, we utilised the MSU to deliver a psychiatric simulation course in Stornoway, Isle of Lewis.

***Description***

A high fidelity mental health simulation course was run for a group of medical students and junior doctors. The course comprised three 15 minute scenarios (a depressed patient, an agitated patient and a distressed relative), followed by a 30-minute debriefing session co-facilitated by 2 experienced psychiatrists. Feedback was collected via anonymous pre and post course questionnaires completed at the time of the course.

***Results & Discussion***

There were 6 participants on the course (3 medical students and 3 junior doctors). When asked about challenges encountered with patients suffering from mental illness in rural areas, participants noted difficulty transferring patients off the island, limited on-site support and the popularity of the area as a destination for patients with mental health problems to travel to.

The majority of participants felt that the course increased their confidence in assessing patients with depression (67%), conducting a suicide risk assessment (83%) and managing agitated patients (83%). All participants noted that the course increased their confidence in using Mental Health Act legislation. Despite the differing levels of the participants, they were all in agreement that the course was appropriate for their learning needs and stage of training. Participants universally agreed that the course increased their preparedness to deal with similar scenarios in a rural healthcare setting.

This was the first time that the MSU has been utilised to deliver mental health simulation to medical students and doctors in rural Scotland, to our knowledge. Participants highlighted specific challenges associated with managing patients with mental illness in remote areas, and valued the opportunity provided by the course to improve their confidence in doing so. The results highlight the importance of improving access to mental health simulation in remote and rural areas.

***Keywords***

mental health, psychiatry, undergraduate, postgraduate, remote, rural

***References***

1. The Scottish Government. People and Communities – Rural Scotland Key Facts 2021. Scotland: Scottish Government; 2021. Available from: https://www.gov.scot/publications/rural-scotland-key-facts-2021/pages/2/.
2. Voluntary Health Scotland. Mental Wellbeing, Social Isolation and Loneliness in Rural Scotland. Scotland: Voluntary Health Scotland; 2019. Available from: https://vhscotland.org.uk/wp-content/uploads/2019/11/Key-Messages-Mental-Wellbeing-in-Rural-Scotland-1.pdf.
3. NHS Education for Scotland. Mobile Skills Unit. Scotland: Clinical Skills Managed Education Network; 2023. Available from: https://www.csmen.scot.nhs.uk/mobile-skills-unit/about/.

## O17. Introducing students to Interventional Radiology through simple Simulation of ultrasound-guided peripheral venous access

### Matthew Gallacher, Nicola Spence, Sarah Noble

#### NHS Highland

*Advances in Simulation 2024*, **9(1):**O17

***Introduction***

Interventional Radiology (IR) is a fast-developing specialty which enables minimally invasive procedures with lower risks than open surgery. Awareness of IR among undergraduates is poor and the authors hope to redress this. Within NHS Highland, limited consultant cover often prevents medical students from attending IR theatre. As a result, a need was identified to give students a simulated introduction to IR in lieu of IR theatre experience. A workshop was therefore designed around ultrasound-guided peripheral venous access; a basic image-guided procedure useful for junior medics. It was delivered to penultimate-year medical students undertaking clinical attachments in Radiology at Raigmore Hospital, Inverness.

***Description***

The session began with slides and video-clips demonstrating (i) the IR theatre environment, (ii) imaging modalities (ultrasound, fluoroscopy) and (iii) Seldinger technique. Ultrasound scanning (USS) technique for locating peripheral veins was then demonstrated on a student volunteer, using an USS machine. This comprised basic elements; sono-anatomy of the upper limb, image optimization and colour Doppler.[1] Students were then invited to use the machine to practise USS technique on one another.

Once students were comfortable with this, a simulation of ultrasound-guided cannulation was demonstrated using an Adam, Rouilly branched 4 vessel vascular access training block model (ABP098), a cannula and the USS machine. Both transverse and in-plane scanning techniques were demonstrated. Students were then invited to perform the same procedure(s) on the model and coached until successful.

***Results & Discussion***

This workshop has taken place four times as of September 2023. Student feedback was very positive; they found the session both interesting and useful, and particularly appreciated the opportunity to learn basic USS technique. Students reported that being able to image the intravascular part of the cannulation process (especially in-plane) aided their understanding of how to successfully site a peripheral venous cannula.

They also said they were more interested in IR following the session, many having never previously heard about the specialty.

As far as we are aware, this is the first time simulation techniques have been used to demonstrate an image-guided procedure within our undergraduate curriculum.[2] The session allowed students to interpret cross-sectional anatomy through ultrasound scanning, and to perform an image-guided procedure through simulated ultrasound-guided cannulation. This is an advanced procedure not conventionally taught until postgraduate level, but was made accessible to medical students through simulation, with positive results.

***Keywords***

Interventional Radiology, Radiology, Simulation, Model, Ultrasound, Ultrasound-Guided, Image-Guided, Imaging, Cannulation, Vascular Access, Undergraduate

***References***

1. Griksaitis MJ, Scott MP, Finn GM. Twelve tips for teaching with ultrasound in the undergraduate curriculum. MedicalTeacher. 2014;36(1):19-24.
2. Patel SG, Benninger B, Mirjalili SA. Integrating ultrasound into modern medical curricula. Clinical Anatomy. 2017;30(4):452-60.

## O18. Leveraging Artificial Intelligence to Support Automated Performance Assessment During Simulation-Based Training

### Scott Pappada^1^. Cristina Alvarado^1^, Kristopher Brickman^1^, Shaza Aouthmany^1^

#### University of Toledo College of Medicine and Life Sciences

*Advances in Simulation 2024*, **9(1):**O18

***Introduction***

Learner proficiency during medical simulation (i.e., operational readiness) is commonly determined using subjective/observational tools such as checklists[1]. These tools tend to reflect measures which have limited detail and are binary in nature (e.g., task was or was not accomplished). Assessment of learners during simulation by an instructor is complicated as the instructor has multiple responsibilities including assessment, directing a scenario, and at times serving as a simulated participant. To this end, even key assessment points or criteria may vary significantly across instructors, be missed, delayed, or suboptimal. There is presently a lack of quantitative and objective performance measures in simulation/medical education. While personalization is crucial to simulation-based medical education effectiveness [2], it is not yet available due to lack of such measures.

***Description***

To address the current limitations in simulation/training, our team has developed a novel assessment platform (PREPARE) to collect subjective and objective data at the learner, instructor, and environmental levels during training activities [3]. Data collection capabilities include subjective instructor assessments, objective physiological markers related to performance from learners wearing wearable devices, and audio data collected from the simulation environment. We have established artificial intelligence (AI) within the platform in the form of speech-to-text (STT) and natural language processing (NLP) of audio data collected within the simulation environment [4]. This capability provides automated detection of interventions/actions/decisions completed during training which can be quantified objectively as timeliness of treatment interventions and clinical decisions. Automated performance assessment capabilities of the platform have currently been validated for stepwise and procedural scenarios such as those requiring advanced cardiac life support (ACLS). The established AI-based capability also provides an added benefit of time synchronizing delayed instructor assessments with physiological data collected from learners. We have observed a correlation between reduced performance and increased stress responses around instructor reported performance measures. The STT and NLP module has been designed such that it accommodates use in other languages outside of English which is important for international collaboration and distribution.

***Results & Discussion***

Platform testing has been completed within procedural tasks and efforts are ongoing to optimize performance during dynamic clinical scenarios. The platform is being leveraged to automate and improve assessment across our undergraduate medical student population during standardized patient encounters. As evaluations can vary significantly amongst different instructors, objective physiological and automated performance measures provide more standardized evaluation metrics. In future use, the platform can be used in real-world clinical settings to detect errors, adverse events, and automatically measure clinical performance.

***Keywords***

automated performance assessment, artificial intelligence, advanced cardiac life support, simulation

***References***

1. Hardy J-B, Gouin A, Damm C, Compère V, Veber B, Dureuil B. The use of a checklist improves anaesthesiologists’technical and non-technical performance for simulated malignant hyperthermia management. Anaesthesia Critical Care & Pain Medicine. 2018;37(1):17-23.
2. Issenberg SB, McGaghie WC, Petrusa ER, Lee Gordon D, Scalese RJ. Features and uses of high-fidelity medicalsimulations that lead to effective learning: a BEME systematic review. Medical teacher. 2005;27(1):10-28.
3. Pappada S, Owais MH, Aouthmany S, Schneiderman J, Toy S, Schiavi A, Miller C, Guris RD, Papadimos T, Rega P. Personalizing simulation-based medical education: the case for novel learning management systems. International Journal of Healthcare Simulation. 2022 Nov 22:1-8.
4. Paudel P, Pappada S, Cheng L. Automated Multimodal Performance Evaluation in Simulation-based MedicalEducation using Natural Language Processing. InProceedings of the ACM/IEEE 14th International Conference on Cyber-Physical Systems (with CPS-IoT Week 2023) 2023 May 9 (pp. 258-259).

**Fig. 1 (abstract O18).**
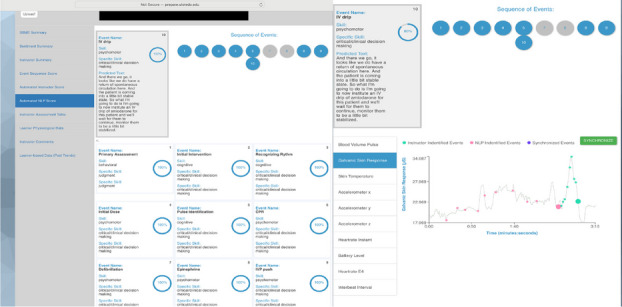
Platform user interface and visualization demonstrating automated performance assessment capabilities (left image) based on real-time detection of interventions during ACLS scenario. Speech to text results for one of the events are represented in each image. Physiological measures (galvanic skin response) collected from simulation participant is shown across the scenario timeline (right image). Note, observer-based instructor performance ratings are distributed at the end of the scenario whereas events detected by the STT and NLP module are detected at millisecond resolution across the scenario timeline. Using the platform, it is possible to synchronize learner physiological responses with each assessment event which provides data related to each learner’s performance and experience.

## O19. Preparing Medical Students to Address Emerging Challenges of Future Wars through Simulation

### Sherri Rudinsky, Rebekah Cole

#### Uniformed Services University

*Advances in Simulation 2024*, **9(1):**O19

***Introduction***

On February 24, 2022, Russia invaded Ukraine, signaling an escalated threat of future large-scale wars. During these wars, both military and civilian physicians may be called to care for significant numbers of patients in large, complex, and violent environments. Medical students should therefore be prepared to practice medicine within challenging wartime environments as a part of their medical school curriculum so that they are ready, if needed, to care for patients wounded in war.

Simulation has been found to play a key role in equipping medical students with clinical skills and abilities needed to care for patients during war. One example of such simulation, the Advanced Combat Medical Experience (ACME), is a 10-day course utilizing prehospital simulations conducted annually at the Uniformed Services University, the United State’s military medical school. ACME simulates a wartime environment, through outdoor high-fidelity prehospital trauma simulations, and introduces second-year military medical students to practicing their prehospital skills amidst the challenges and obstacles of war. No studies currently exist examining the benefits of prehospital simulation to prepare junior medical students for future wars. Our study, therefore, examined the experiences of second-year medical students during ACME and their personal and professional development while caring for patients during a simulated wartime environment.

***Methods***

We used the phenomenological tradition of qualitative research to analyze the experiences of students who participated in ACME. To explore their experiences, our research team closely reviewed the post-simulation reflection papers written by 176 second-year medical students who attended ACME during summer 2021. We then coded each paper and came to a consensus on the themes and patterns within the data, which served as the results of our study. We bracketed our biases throughout the data analysis process in order to enhance the validity of our results.

***Results & Discussion***

We discovered four themes within the data illustrating the students’ experiences during ACME: 1) self-confidence, 2) teamwork, 3) stress management, and 4) professional identity formation. The students became more confident in their ability to practice medicine during an out of hospital wartime environment. While they were at first surprised by how hard it was to work as a team, the students learned to trust their teammates in order to overcome challenges. They also learned how to manage their stress by grounding themselves and relying on their teammates. Finally, after successfully completing ACME, the students were able to envision themselves as military physicians, affirmed in their career path. Based on these results, ACME may serve as a model for using simulation to prepare both military and civilian physicians to care for patients amidst the emerging challenges of wartime environments and contributes to better understanding the benefits of incorporating environmental fidelity in healthcare simulation.

***Keywords***

Trauma Simulation, War, professional identity formation, environmental fidelity

***References***

Rudinsky SL, Weissbrod E, Cole R. “Not for the Faint of Heart”: First-year Military Medical Students’ Professional Identity Formation During the Innovative Patient Experience at Operation Bushmaster. Military Medicine. 2023;188(Supplement_3):34–40.
Rudinsky SL, Weissbrod E, Cole R. The Impact of the Patient Role on Medical Student Learning During Peer Simulation. Simulation in Healthcare: The Journal of the Society for Simulation in Healthcare. 2022;Publish Ahead of Print.
Rudinsky SL, Spalding C, Conley SP, Everett L, Cole R. The development, implementation, and evaluation of a medical student peer teaching training curriculum during a high fidelity prehospital trauma simulation. AEM Education and Training. 2022;6(4).
4.Cole R, Rudinsky SL, Conley SP, Vojta L, Kwon SW, Garrigan AG, et al. The Impact of Medical School on Military Physicians’ Readiness for their First Deployment. Military Medicine. 2022 Mar 8;187(7-8):e995–1006.
Creswell JW. Qualitative inquiry & research design : Choosing among five approaches. 3rd ed. Los Angeles: Sage Publications; 2013. DoD Disclaimer: “The opinions and assertions expressed herein are those of the author(s) and do not reflect the official policy or position of the Uniformed Services University of the Health Sciences or the Department of Defense.”

## O20. Simulation as part of a complex and entangled puzzle

### Jane Hislop^1^, Tom Fawns^2^, Nathan Oliver^3^, Xinchi Zhang^1^, Susan Somerville^4^

#### ^1^University of Edinburgh, ^2^Monash University, ^3^University of Canberra, ^4^University of Dundee

*Advances in Simulation 2024*, **9(1):**O20

***Introduction***

Simulation-based education (SBE) is “massively on the rise, highly technological, but under-theorised” (p. 905) [1].

Discussions often focus on methods and technologies [2, 3] seeing SBE sessions as an adjunct to existing curricula [4]. Recent research highlights challenges of embedding simulation into a broader healthcare landscape [5] that may limit its effectiveness. . Our project seeks to understood how SBE fits alongside other learning (e.g. clinical teaching) and how it is affected by a combination of factors within a wider education ecosystem.

Our ambitious study seeks to shed new light and fresh insights into the complex, sociomaterial landscape of simulation in healthcare environments. Focusing on simulation sessions across two Scottish medical schools, we are exploring how these sessions are situated in wider contexts, where diverse purposes and values are in play.

***Methods***

Using an entangled simulation framework (figure 1), this focused ethnographic study [7] involved analysis of documents (including policies, curriculum specifications, lesson plans, and simulation artefacts), observation of simulation facilitators and participants from a backstage and front of house perspective, and unstructured interviews with stakeholders, facilitators, and trainees at simulation centres in Edinburgh and Dundee. The project was funded by the Scottish Medical Education Research Consortium and has ethical approval from the University of Edinburgh, Medical Education Research Ethics Committee.

***Results & Discussion***

Preliminary results suggest that what happens in these simulation sessions is a combination of carefully planned, arbitrary, and emergent factors, that are contingent on competing priorities and a mix of strategic and ad hoc configurations of infrastructure. Facilitators use a blend of knowledgeable and ad hoc practices to negotiate this complexity into educational experiences that are relevant and meaningful, even if these are not exactly as described in design and policy documents. What happens within simulation is more loosely coupled with pre-specified outcomes than is suggested within simulation discourse and documentation, but this looser coupling allows for valuable, emergent and unanticipated experiences. The relevance of these experiences to other learning events is contingent on factors both within and beyond the simulation scenarios and debrief sessions.

Our research is highlighting considerations beyond the design of simulation sessions that influence the activity that happens within sessions and the relevance of that activity to other learning events. Such considerations are important to curriculum planning in which simulation is seen as just one aspect of a medical trainee’s learning journey.

***Keywords***

N/A

***References***

1. Johnston JL, Kearney GP, Gormley GJ, Reid H. Into the uncanny valley: Simulation versus simulacrum? Med Educ. 2020; 54(10): 903–7.
2. Issenberg SB, Ringsted C, Øostergaard D, Dieckmann P. Setting a research agenda for simulation-based healthcareeducation a synthesis of the outcome from an utstein style meeting. Simul Healthc. 2011; 6(3): 155–67.
3. Battista A, Nestel D. Simulation in Medical Education. In: Understanding Medical Education: Evidence, Theory, andPractice. Oxford: Wiley-Blackwell; 2019. pp. 151–62.
4. Gaba DM. The future vision of simulation in health care. Qual Saf Heal Care. 2004; 13(SUPPL. 1): 2–10.
5. Davies, E., Montagu, A. & Brazil, V. Recommendations for embedding simulation in health services. Adv Simul. 2023; 8, 23. https://doi.org/10.1186/s41077-023-00262-3
6. Fawns T, Hislop J, Oliver N, Somerville S. An entangled view of an educational activity. 2022. Availablefrom: https://open.ed.ac.uk/an-entangled-view-of-an-educational-activity.
7. Vindrola-Padros C. (2020) Team-based Rapid Ethnographies in Vindrola-Padros C., Rapid Ethnographies A PracticalGuide Cambridge University Press. https://doi-org.ezproxy.is.ed.ac.uk/10.1017/9781108623568

**Fig. 1 (abstract O20).**
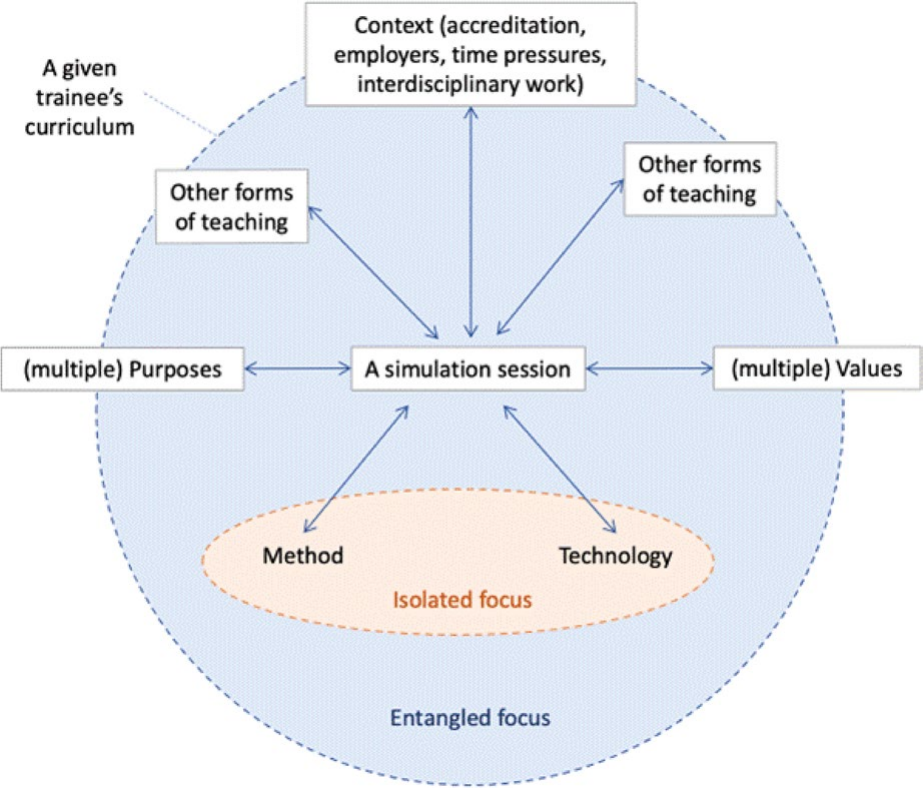
See text for description

## O21. Stepping Back for Safety – A simulation-based intervention for adaptive expertise

### Maria Louise Gamborg^1^, Kasper Glerup Lauridsen^1^, Cirkeline Hytte Pedersen^1^, Peter Dieckmann^2^, Kristian Brogaard Krogh^3^, Mads Lind Ingeman^3^, Yoon Frederiksen^1^, Maria Mylopoulos^4^

#### ^1^Aarhus University, ^2^University of Copenhagen, ^3^Aarhus University Hospital, ^4^University of Toronto

*Advances in Simulation 2024*, **9(1):**O21

***Introduction***

Clinical decision-making (CDM) is a key competence for physicians that refers to the ability to identify, sort, and prioritize relevant information, with the aim of deciding on a specific diagnosis (1, 2). It is a process that involves a lot of human cognitive processing which is under pressure in the healthcare setting. Disruptions often occur in the clinical setting due to e.g. technical issues with the equipment, relatives demanding attention, or multiple sick patients causing attention (3, 4). Such disruptions increase the demand on cognitive processing and may affect CDM. Adaptive Expertise (AE) encompass cognitive strategies that promote flexible, creative and innovative use of knowledge, to solve novel and complex problems. Therefore, AE has been proposed as a highly relevant approach to CDM, as it prompts clinicians to take a step back to critically reflect on their knowledge (5, 6). Despite the potential to train these essential cognitive competencies exists, and could reduce risk for patient, no educational initiative targets the challenge of handling disruptions. Here, simulation-based education can serve as a unique mode of training. Thus, this study aimed to investigate the psychological impact of two different disruptions during medical emergencies.

***Methods***

We designed an experimental study to qualitatively investigate the impact of four conditions: (1) environmental disruptions (faulty equipment), (2) psychological disruptions (treating two patients at once), (3) the combined disruptions, and (4) the simulation without disruptions (control). Participants are randomized to one condition, resulting in 5 participants in each. Directly after the training, participants take part in a semi-structured interview. All interviews are transcribed and analyzed through reflective thematic analysis, using the adaptive expert framework as a sensitizing concept.

***Results & Discussion***

In all, 20 post-graduate year 1 doctors will be recruited. Data collection is still ongoing, and all data will be collected and analyzed before SESAM 2024, where results will be presented. Preliminary results indicate that the psychological disruptions, either on their own or in combination with an environmental disruption, was experienced as highly unexpected and disruptive to their decision-making process. Particularly themes of entrusting others with acting out one’s decisions was identified as a difficult element to overcome. Preliminary reports from the included participants describe that the psychological disruption prompted more internal struggle, leading to creative and innovative use of knowledge. Final conclusions will be presented at the conference.

***Keywords***

Clinical decision-making, Adaptive expertise, disruptions, emergency medicine

***References***

1. Croskerry P. Clinical decision making. Pediatric and Congenital Cardiac Care: Volume 2: Quality Improvement andPatient Safety2015. p. 397-410.
2. Croskerry P. ED cognition: any decision by anyone at any time. Canadian Journal of Emergency Medicine. 2014;16(1):13-9.
3. Gamborg ML, Mehlsen M, Paltved C, Vetter SS, Musaeus P. Clinical decision-making and adaptive expertise inresidency: a think-aloud study. BMC Medical Education. 2023;23(1):22.
4. Gamborg ML, Mylopoulos M, Mehlsen M, Paltved C, Musaeus P. Exploring adaptive expertise in residency: the(missed) opportunity of uncertainty. Advances in Health Sciences Education. 2023.
5. Hatano G, Inagaki K. Two Courses of expertise. In: Stevenson H, Azuma H, Hakuta K, editors. Child Developmentand Education in Japan. New York: WH Freeman; 1986. p. 27-36.
6. Branzetti J, Gisondi MA, Hopson LR, Regan L. Adaptive expertise: The optimal outcome of emergency medicinetraining. AEM Educ Train. 2022;6:e10731.

## O22. Student Survey Results from a Pan-Canadian Virtual Simulation Program

### Margaret Verkuyl^1^, Lynda Atack^1^, Efrem Violato^2^, Nicole Harder^3^, Theresa Southam^4^, Melanie Lavoie-Tremblay^5^, Sandra Goldsworthy^6^, Wendy Ellis^7^, Suzanne Campbell^8^

#### ^1^Centennial College, ^2^North Alberta Institute of Technology, ^3^Unversity of Manitoba, ^4^Selkirk College, ^5^University of Montreal, ^6^Mount Royal University, ^7^George Brown College, ^8^The University of British Columbia

*Advances in Simulation 2024*, **9(1):**O22

***Introduction***

A global shortage of healthcare providers has made it increasingly difficult for healthcare educators to secure quality clinical placements. The Virtu-WIL program, a pan-Canadian initiative, was designed for to develop, offer, and test over 100 work-integrated virtual simulations for healthcare students to alleviate this gap. Nursing, medical laboratory technologist and paramedicine students participated in the program.

***Methods***

A mixed-methods evaluation, guided by Kirkpatrick’s model, was conducted to explore students’ experiences with the virtual simulations and, importantly, to determine the impact of the simulations on their clinical practice. Students reported their experiences using a survey that included validated subscales and open-ended items. Data were analyzed using descriptive and inferential statistics. Open-ended comments were analyzed for themes using a process described by Braun and Clarke (2006).

***Results & Discussion***

1715 students enrolled in the Virtu-WIL program from 18 post-secondary universities, colleges and institutions across Canada completed the survey. Satisfaction with the virtual simulations was high (86.2%). The mean score for the functionality and engagement subscale was 82/100 suggesting students found the virtual simulations easy to use and aesthetically and intrinsically appealing. Students reported that the simulations provided realistic, meaningful, immersive experiences which contributed to their learning and strongly contributed to students’ perceptions of readiness for practice. The virtual simulations provided an opportunity for students to see their professional roles in action and to take on that role. Students had the chance to think things through, make mistakes and learn from their mistakes in a safe environment.

Virtual simulation has emerged as a learning modality that can help students develop key employability skills. The results of this pan-Canadian project, with students from three different healthcare programs found that the virtual simulations were engaging, helped students to learn and prepare for practice. A key finding was that it is not sufficient to simply add virtual simulations to the curriculum, careful alignment with course objectives and simulation pedagogy are essential. We anticipate that the findings from this large, multi-site evaluation will be of interest to educators from a range of programs who are interested in adopting virtual simulation in education. We will add that the virtual simulation experiences used in this project are freely available through creative commons licensing to the global health care community at https://simulationcanada.ca/virtu-wil/.

***Keywords***

virtual simulation, program evaluation, Pan-Canadian, survey

***Reference***

1. Braun, V., & Clarke, V. Using thematic analysis in psychology. Qualitative Research in Psychology, 2006: 3, 77-101. 10.1911/478088706qp063oa.

## O23. Sustainable Novel 3D Alginate Models for Simulation-Based Medical Education in Pathology: A Validation Study

### Eduardo Alcaraz-Mateos^1^, Candela Salmeron-Lopez^2^, Juan Francisco Minarro-Jimenez^2^, Angeles Abellan-Palazon^1^, Fuensanta Caballero-Aleman^1^, Nicolas Sanchez-Campoy^3^, Kamran M Mirza^4^, Xiaoyin Sara Jiang^5^

#### ^1^Morales Meseguer University Hospital, ^2^University of Murcia, ^3^INE - National Statistics Institute, ^4^Loyola University Medical Center, ^5^Duke University Hospital

*Advances in Simulation 2024*, **9(1):**O23

***Introduction***

Alternatives to formaldehyde-fixed and fresh specimens are a convenient solution for simulation-based pathology education for medical students, pathologist assistants and trainee pathologists (1). Alginate is economical, vegan, and biodegradable, providing a sustainable alternative to other materials such as gelatin and silicone. The aim of this study was to validate novel custom-made alginate-based 3D models for macroscopic examination or gross dissection training.

***Methods***

Specimens were hand-built, scanned with the EinScan Pro 2X Plus, and modeled into 3D printed molds using SolidWorks software and the Stratasys F170 with ABS filament. Using these molds, polypectomy, thyroidectomy and skin resection specimens made of alginate were created. These models were replicated and distributed to pathology educators across the country. A 5-point Likert-style questionnaire through Google forms was supplied for face and content validity, considering aspects such as realism, consistency, color and size, ability to be inked, behavior to the section, to assess the utility of the models.

***Results & Discussion***

37 teachers (8% professors, 19% associate professors, and 73% assistant/adjunt professors) from different institutions (12 hospitals belonging to 9 universities in 7 cities) participated in this 3-week study.

Face validity was demonstrated with overall average teachers' scores of 4.39 out of 5 (range 3-5, SD:0.72). Best results were for the skin resection (4.59), followed by thyroid (4.49) and polyp models (4.08). Content validity or educational value was also proved: all participants agreed that the models allowed effective teaching of pathologic anatomy (4.59), neoplastic pathology (4.27), grossing (4.81), surgical margin concepts (4.92), and hand-eye coordination (4.86). Professors' age was positively correlated with the utility perceived, so that the older the age of the faculty, the greater the utility considered of the models (*p*<0.05).

These new alginate-based models were shown to have face and content validity for the three common gross specimens tested, when evaluated by pathologist faculty educators from a variety of institutions and faculty ranks.

Interestingly, the most experienced professors were the ones who perceived the most value to these models. The novel alginate-based models offer a sustainable alternative to existing materials for teaching macroscopic examination. These results demonstrate efficacy of these models for a standardized teaching in an interactive way, with skills acquisition among trainees.

***Keywords***

Pathology; Dissection; Grossing; Macroscopic examination; 3D printing; Additive manufacturing

***Reference***

1. The utility of a gross dissection anatomical model for simulation-based learning in pathology. Alcaraz-Mateos E, Mirza KM, Molina-Valverde S, Togkaridou M, Caballero-Alemán F, Poblet E. Rev Esp Patol. 2022;55(3):149-155.

**Fig. 1 (abstract O23).**
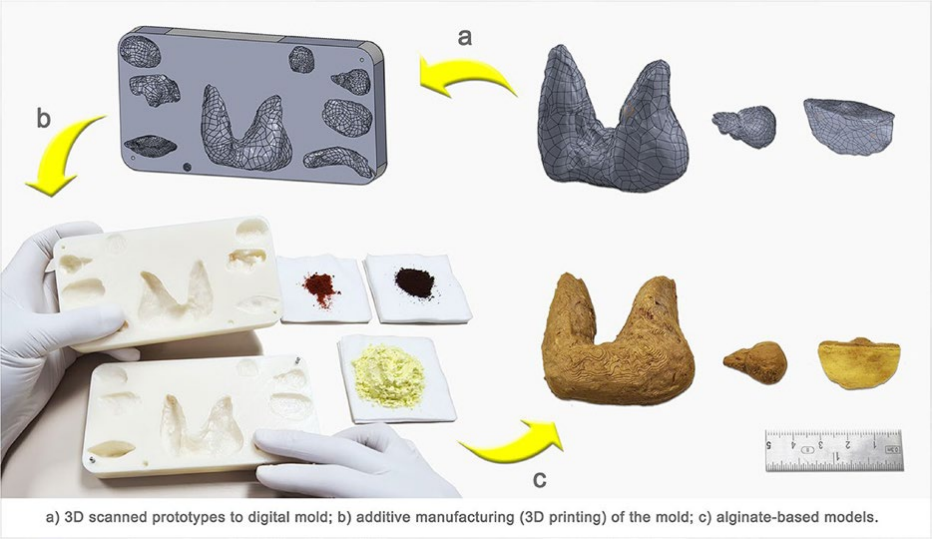
**a** 3D scanned prototypes to digital mold; **b** additive manufacturing (3D printing) of the mold; **c** alginate-based models

## O24. Think GP: Utilising simulation to enhance General Practice teaching in undergraduate education

### Robyn Gatenby^1^, Joanna Traynor^1^, Sharon Donaghy^1^, Sarah Luty^2^

#### ^1^NHS Lanarkshire, ^2^NHS Education for Scotland

*Advances in Simulation 2024*, **9(1):**O24

***Introduction***

There is a crisis of General Practitioner (GP) recruitment, with GP numbers falling (1). To try to combat this, medical student places are being increased across the country, and the government have mandated that 50% of graduates pursue a career in general practice (2). Experience in undergraduate general practice is directly related to subsequent career choice (3,4). Therefore, there is a target for at least 25% of undergraduate clinical teaching to be in general practice (5). With reducing numbers of full time equivalent (FTE) GPs and ever-increasing workload, finding higher numbers of student placements is a challenge (6).

As a result, we piloted and evaluated a GP simulation course with forty third year undergraduates at University of Glasgow.

Our aim was to increase students’ exposure to general practice, and allow them to understand the challenges and uniqueness, including the limitations of managing patients who present with acute illness.

***Description***

We utilised a course we had previously developed for doctors in their first year of GP training and adapted these scenarios to be suitable for undergraduate level. The scenarios varied in age, culture and medical specialty to highlight the breadth of general practice. Similar scenarios are used in other simulation courses the students experience in secondary care and highlighted the differences in environmental factors and how this influenced decision making.

Students were supported by faculty confederate e.g. practice nurse in the room.

- Scenario 1 - middle aged diabetic patient with hypoglycaemia during Ramadan.
- Scenario 2 - adult patient walk in with anaphylaxis.
- Scenario 3 - 10 month old baby with meningitis.
- Scenario 4 - depressed patient with suicidal thoughts.
- We utilised pre and post course questionnaires to assess how this course impacted on various areas.

***Results & Discussion***

The feedback was positive, with confidence scores increasing between pre- and post-course evaluation in all twelve domains, including technical and non-technical skills. On asking students if they would consider general practice as a career, one out of eleven who had said they would not, changed their mind and said they would now consider being a GP. Qualitative data was very positive. The data shows that students had a positive experience with improved confidence in consulting in the general practice setting, and that it may encourage students to consider a career in general practice who were not previously considering this. This may be able to be extrapolated to other university medical students across the country.

***Keywords***

General Practice, Undergraduate experience, Environmental factors

***References***

1. Marchand C, Peckham S. Addressing the crisis of GP recruitment and retention: a systematic review. British journal of general practice 2017;67(657):e227-e237.
2. Department of Health. Delivering high quality, effective, compassionate care: developing the right people with theright skills and the right values: A Mandate from the Government to Health Education England. 2013.
3. Alberti H, Randles HL, Harding A, McKinley RK. Exposure of undergraduates to authentic GP teaching andsubsequent entry to GP training: a quantitative study of UK medical schools. British journal of general practice 2017;67(657):e248-e252.
4. Blythe A. Teaching general practice: a rallying flag for undergraduate education. British journal of general practice. 2018;68(677):560-561.
5. Harding A, Hawthorne K, Rosenthal J. Teaching general practice: Guiding principles for undergraduate generalpractice curricula in UK medical schools. 2018.
6. Harding A, Rosenthal J, Al-Seaidy M, Gray DP, McKinley RK. Provision of medical student teaching in UK generalpractices: a cross-sectional questionnaire study. British Journal of General Practice 2015;65(635):e409-e417.

## O25. Use of extended reality (XR) tools: an adjunct for undergraduate neuroanatomy education

### Anna Moore^1^, Brooke Nash^1^, Darcey Atkinson^1^, Rosalia Nash^1^, Sameera Hettipathirannahelag^2^, Jack Sheppard^2^, Latif Miah^2^, Clinton Mitchell^2^, Owen Crawford^3^

#### ^1^School of Medicine, ^2^Department of Neurosciences, ^3^Cardiff Learning & Teaching Academy

*Advances in Simulation 2024*, **9(1):**O25

***Introduction***

Neuroanatomical knowledge is vital when assessing patients with neurological conditions, yet poses educational challenges. Using Extended Reality (XR) [combining immersive reality (IR); augmented reality (AR); and virtual reality (VR)] for anatomy teaching is gaining popularity within medical education (1, 2). We aimed to evaluate the effectiveness of XR adjuncts along with near-peer neuroanatomy teaching of undergraduate medical students in enhancing student spatial ability (3).

***Description***

IR (projection room displaying 3D-anatomical models), AR (overlaying AR structures onto a brain model) and VR (traversal and manipulation of 3D-neuroanatomical models via Oculus headset) were integrated into small-group sessions (Figure-1) delivered to 29 fourth-year medical students (Mean age= 22.3 years; M:F ratio=1: 2.9) by near-peers (fifth-year medical students).

A service evaluation was performed using anonymised pre- and post-session feedback. A five-point Likert scale [very unconfident(score=1) to very confident(score=5)] determined change in student confidence with neuroanatomy. Pre- and post-session single-best-answer questions (SBAs), consisting of 6 image-based questions (IBQs) assessing neuroanatomical spatial ability and 6 text-based questions (TBQs) assessing neuroanatomical factual recall, were used to objectively evaluate knowledge. Change in student performance in IBQs versus TBQs pre- and post-session was determined. Statistical significance was calculated using Wilcoxon ranking and Mann-Whitney U tests. Data collection ongoing.

***Results & Discussion***

Most students (63%, *n*=29) found combining XR modalities of maximum benefit. 72% (*n*=29) deemed the near-peer session more useful and felt more comfortable asking questions compared to clinician-delivered sessions. Post-session, students were more confident in identifying neuroanatomical structures [Pre-session median=3.00 (Interquartile range (IQR)=3.00-1.00) vs Post-session median=4.00 (IQR =4.00-1.00), *p*<0.001] and recalling neuroanatomical facts [Pre-session median=2.00 (IQR=2.00-1.00) vs Post-session median=4.00 (IQR=2.00-0.00), *p*<0.001]. Performance in neuroanatomy SBAs significantly improved for IBQs [Pre-session mean=2.411.15 standard deviation (SD) vs Post-session mean=3.801.15 SD, *p*<0.001] and TBQs [Pre-session mean=3.241.15 SD vs Post-session mean=4.071.19 SD, *p*=0.001].

Pre-session, students performed better in TBQs than IBQs (Mean TBQ score=3.241.15 SD vs Mean IBQ

score=2.411.15, *p*=0.009), but there was no significant difference post-session (Mean TBQ score=4.071.19 SD vs Mean IBQ score=3.801.15, *p*=0.197).

This suggests that our XR session particularly addressed deficits within visuospatial applications of neuroanatomical knowledge. The closure of this gap by XR teaching may have significant implications for the provision and modality of medical education in specialties with a propensity for visuospatial problems, such as surgery, pathology and radiology.

***Keywords***

Clinical Neuroscience; Extended reality; Integrated reality; Augmented reality; Undergraduate; Medical Education; Near-Peer; Neuroanatomy; Neurosurgery; Neurology; Neuroradiology

***References***

1. Scantling-Birch Y, Naveed H, Tollemache N, Gounder P, Rajak S. Is undergraduate ophthalmology teaching in theUnited Kingdom still fit for purpose? Eye (London, England). 2022;36(2):343-5.
2. Hettipathirannahelage S, Miah L, Sheppard J, Mitchell C. Utilizing virtual reality to train medical students to identifyrelative afferent pupillary defects [abstract]. In: IPSSW2023, Improving Pediatric Outcomes with Simulation: Human Factors and Technology: 2023 May 17-19; Lisbon, Portugal.
3. Uttal DH, Meadow NG, Tipton E, Hand LL, Alden AR, Warren C, et al. The malleability of spatial skills: ameta-analysis of training studies. Psychol Bull. 2013;139(2):352-402.

## O26. Using Virtual Simulations to Promote Global Health Equity, Diversity, and Inclusion

### Marian Luctkar-Flude^1^, Jane Tyerman^2^

#### ^1^Queen's University, ^2^University of Ottawa

*Advances in Simulation 2024*, **9(1):**O26

***Introduction***

A major barrier to global health equity is resource limitations including lack of access to educational resources and health equity training.(1-3) There is a need for accessible resources for health professionals and students to learn and stay up to date with evidence-based clinical practices and guidelines. Additionally, persons who identify as racial, sexual or gender minorities experience health inequities due to discrimination. Thus, there is a need for education for health professionals and students about practicing with cultural humility, and a need to provide resources to underrepresented persons and allies to address microaggressions in clinical settings. There is also a global need for accessible and effective educational interventions for patients with chronic diseases to promote self-management. Virtual simulations are screen-based simulations that can be used to address these gaps. We aim to describe development and use of a series of virtual simulations for clinical education, equity, diversity, and inclusion (EDI) education, and patient self-management education to promote global health equity.

***Description***

Over the past five years, a group of nurse educators from Canada collaborated on a series of online learning modules incorporating virtual simulations. We will share a cost-effective and user-friendly process for nurse educators to create virtual simulations that address educational needs of healthcare providers, learners, and patients. We will highlight virtual simulations we created about (i) addressing racism and microaggressions in clinical and classroom settings, (ii) providing culturally humble care to sexual and gender diverse individuals, (iii) wound care for persons with different skin tones, and (iv) patient self-management education in low-resource countries (Table 1). Our modules include presimulation preparation, debriefing strategies, and resources in alignment with the Healthcare Simulation Standards of Best Practice.(4)

***Results & Discussion***

Our project has demonstrated that virtual simulations are an effective educational strategy for health professionals, students, and patients. Virtual simulations are a novel and cost-effective method to create clinical, EDI, and patient self-management education content that can be shared globally.(5) Collaborations between simulation experts, content experts and persons with lived experience have contributed to the authenticity and value of eLearning modules. Our EDI virtual simulations are hosted open-access and many of these simulations have been accessed over 3 million times by users from around the world, providing evidence of the reach and impact of this project. Virtual simulations can be used to promote global health equity by increasing accessibility to evidence-based healthcare education and patient education in low-resource regions and institutions.

***Keywords***

healthcare education; patient education; virtual simulations; health equity; equity, diversity and inclusion;

***References***

1. Frenk J, Chen LC, Chandran L, Groff EOH, King R, Meleis A, et al. Challenges and opportunities for educating healthprofessionals after the COVID-19 pandemic. Lancet. 2022;400(10362):1539-56.
2. Hojat LS. Breaking down the barriers to health equity. Ther Adv Infect Dis. 2022;9:20499361221079453.
3. Landry AM. Integrating health equity content into health professions educatoin. AMA Journal of Ethics. 2021;23(3):E229-E34.
4. Watts PI, McDermott DS, Alinier G, Charnetski M, Ludlow J, Horsley E, et al. Healthcare Simulation Standards of BestPractice^TM^ Simulation Design. Clinical Simulation In Nursing. 2021;58:14-21.
5. Tyerman JL-F, M.; Chumbley,L.; Lalonde,M.; Peachey,L.; Tregunno,D. Developing virtual simulation games forpresimulation preparation: A user-friendly approach for nurse educators. Journal of Nursing Education and Practice. 2021;11(7):10-8.

**Table 1 (abstract O26).** Summary of Open-Access Virtual Simulations to Promote Global Health Equity

Online Learning Modules	Target Audience	Virtual Simulations
**Responding to Racism** https://can-sim.ca/responding-to-racism/	Health Professional Students	**• Differentiating between Overt and Covert Racism:** 5 mini-scenarios **• Clement’s Story:** a Black student experiences racism in the clinical setting **• Allie’s Story:** a white student witnesses racism in the classroom
**Sexual Orientation and Gender Identity (SOGI) Nursing** https://soginursing.ca/	Health Professionals and Students (Available in English and French)	**• Wolfgang’s Story:** an older gay man experiences loss of a spouse **• Cody’s Story:** a transgender youth experiences anxiety **• Sarah’s Story:** a queer female experiences gender assumptions **• Connor’s Story:** a transgender man experiences misnaming **• Additional Mini-Simulations**
**Wound Assessment and Management** https://woundnursing.ca/	Health Professionals and Students	**• Pre and Post Virtual Simulation Quizzes** **• Clara’s Story:** older woman with a pressure injury **• Adam’s Story:** younger man with a venous leg ulcer **• Ted’s Story:** middle-aged man with a diabetic foot ulcer **• Tesfaye’s Story:** a recent immigrant from Ethiopia with an infected incision
**Diabetic Foot Care Education** http://www.can-sim.ca/games/adfc/story_html5.html	Patients, Health Professionals and Students from Ethiopia (Available in Amharic language)	**• Diabetic Foot Care Education in Ethiopia**

## O27. Virtual Reality as a Self-Paced Learning Approach for Continuous Renal Replacement Therapy System Set-up

### Pedro Cartaxo Cintra^1^, Miquel Sanz Moncusi^1^, Laura Lorenzo Montesinos^2^, Christian Rodriguez Costoya^2^, Laia Zamora Masso^2^, Iago Enjo Perez^3^, Esther Leon-Castelao^3^, Maurício Olivares Rojas^3^, Dr. Jose Maria Nicolas Arfelis^1^

#### ^1^Hospital Clínic de Barcelona - Universitat de Barcelona, ^2^Hospital Clínic de Barcelona, ^3^Universitat de Barcelona

*Advances in Simulation 2024*, **9(1):**O27

***Introduction***

Continuous renal replacement therapies (CRRT) are vital in critical care, and healthcare professionals must be proficient in their set-up and management to ensure improved patient outcomes. Specialized training is essential due to the complexity of CRRT initiation1. However, creating an effective training program for CRRT set-up presents challenges, including procedure complexity, time constraints, and variations in device brands and settings. Current simulation-based educational strategies are time-consuming and resource-intensive, making the development of a self-paced learning environment using virtual reality (VR) an attractive alternative2,3. This study aims to evaluate the educational impact and feasibility of this novel approach.

***Methods***

A quasi-experimental study with pre-post intervention measurements was conducted at the University of Barcelona and

Hospital Clínic of Barcelona4. Physicians and registered nurses engaged in an interactive step-by-step VR scenario of Baxter Prismax▪ CRRT device. Before VR exposure, participants completed a CRRT set-up-related ad hoc questionnaire.

The intervention involved an interactive VR scenario simulating a critical care unit, guiding participants through the CRRT device set-up process, emphasizing material selection, placement, and recommendations while recording the time. The prebriefing, scenario, and feedback processes were self-paced through the VR. The investigators provided technical support but did not interfere in this process, and the scenario could only be run once.

After the intervention, participants once again completed the earlier questionnaire, the System Usability Scale▪5, and the Simulator Sickness Questionnaire▪6. Real CRRT device set-up was performed immediately after the intervention and at 3, 6, and 12-month intervals, with an expert directly measuring the participants' performance.

***Results & Discussion***

The study included 54 healthcare professionals. Preliminary results indicate that each professional completed the VR scenario in an average of 26 minutes (± 6’) with minor errors during the immediate real CRRT device set-up, highlighting the time benefits of the VR self-paced learning strategy.

The Simulator Sickness Questionnaire▪ indicated issues with blurry vision (possibly due to VR device positioning), and major side effects like nausea or sickness were not frequently reported. Additionally, the System Usability Scale▪ assessment rated the strategy as user-friendly, with an efficiency, capability, and functionality level of A-, surpassing acceptable standards. The three final measurement periods are still pending.

These initial findings suggest that VR may be a suitable self-paced learning approach for healthcare professionals to develop the skills required for CRRT device set-up. Further measurements are needed, and a comparison with previous simulation-based education strategies is necessary to evaluate the overall impact and benefits of this approach.

***Keywords***

Virtual reality; Continuous renal replacement therapies; Simulation-based education

***References***

1. Joannes-Boyau O, Velly L, Ichai C. Optimizing continuous renal replacement therapy in the ICU: A team strategy. Vol. 24, Current Opinion in Critical Care. Lippincott Williams and Wilkins; 2018. p. 476–82.
2. Lemarie P, Husser Vidal S, Gergaud S, Verger X, Rineau E, Berton J, et al. High-Fidelity Simulation Nurse Training Reduces Unplanned Interruption of Continuous Renal Replacement Therapy Sessions in Critically Ill Patients: The SimHeR Randomized Controlled Trial. Anesth Analg. 2019;129(1):121–8.
3. Macnamara AF, Bird K, Rigby A, Sathyapalan T, Hepburn D. High-fidelity simulation and virtual reality: An evaluationof medical students’ experiences. BMJ Simul Technol Enhanc Learn. 2021;7(6):528–35.
4. Hospital Clínic de Barcelona. Ethics Committee Approval - Dictamen - Impacto de la Realidad Virtual en el Montaje deCircuitos de Terapias Continuas de Reemplazo Renal. Spain: HCB/2023/0373; 2023.
5. Sevilla-Gonzalez MDR, Moreno Loaeza L, Lazaro-Carrera LS, Bourguet Ramirez B, Vázquez Rodríguez A,Peralta-Pedrero ML, et al. Spanish Version of the System Usability Scale for the Assessment of Electronic Tools: Development and Validation. JMIR Hum Factors. 2020;7(4):e21161. Available from: http://humanfactors.jmir.org/2020/4/e21161/.
6. Campo-Prieto P, Rodríguez-Fuentes G, Ma Cancela-Carral J. Traducción y adaptación transcultural al español del Simulator Sickness Questionnaire Translation and cross-cultural adaptation to Spanish of the Simulator Sickness Questionnaire. Vol. 43. Available from: https://recyt.fecyt.es/index.php/retos/index

## O28. “Don’t talk to me, I’m busy operating”: Does good teamwork and surgical performance require much talking?

### Rune Dall Jensen, Amalie Asmind Rosendal, Ita Klingenberg, Maria Louise Gamborg, Anders Schram

#### Aarhus University

*Advances in Simulation 2024*, **9(1):**O28

***Introduction***

Recent research highlights that human motor skills are inherently noisy, which affects the stability of motor skill performance (1). In essence, surgical technical skills are regarded as cognitive skills. According to cognitive load theory, these technical skills may be impacted the quantity and quality of communication and teamwork. Thus, the performance of technical skills is intertwined with the non-technical skills of managing the operating room (OR). However, most simulation-based studies tend to separate technical and non-technical skills within the OR, leaving the influence of communication quality on teamwork and technical performance unexplored. This study aims to investigate how the primary surgeon's communication with the OR team affects both technical performance and teamwork.

***Methods***

We created team-based simulated scenarios on live animal tissue. Each team consisted of two surgeons in specialist training and two OR nurses, who together performed 12 full surgical procedures spaced out on three days. Each scenario was recorded, and the primary surgeon’s communication was investigated using descriptive analysis and Spearman Correlation Analysis. The amount of verbal communication was compared with the performance on both technical and non-technical skills. These outcomes were assessed by 5 blinded raters on Non-technical Skills for Surgeons (NOTSS) (2) and Objective structured assessment of technical skill (OSATS) (3).

***Results & Discussion***

In total, 3 teams and 54 videos were analysed. There was a medium positive correlation between surgical teams’ NOTSS and OSATS scores (*r* =0,39, *p* =,004). Verbal communication by the primary surgeon showed a medium correlation with NOTSS scores (*r*= 0,33, *p* = ,015) but no correlation to OSATS (*r*= 0,12, *p* = ,389) scores. This indicates that teamwork and surgical performance is not equally influenced by the amount of speaking between team leader and other team members. This might show that teamwork is independent of how much the team leader perform verbal communication and that non-verbal communication is of high importance. Another interpretation is that the subjective assessment of non-technical skills is not dependent on quantity of verbal communication. In summary, the findings indicate that non-technical skills are positively correlated with technical skills, and that the quantity of verbal communication may have a moderate influence on non-technical skills but does not significantly affect technical skills. It also raises questions about the ability of the NOTSS tool to distinguish between the quantity and quality of verbal communication, while it seems as the NOTTS scores is more than the sum of its parts.

***Keyword***

Surgery

***References***

1. Jensen RD, Brydges R, Grierson L. Re-examining the integration of routine and adaptive expertise: there is no suchthing as routine from a motor control perspective. Adv Heal Sci Educ. 2022;27(5):1283–91.
2. Yule S, Flin R, Maran N, Rowley D, Youngson G, Paterson-Brown S. Surgeons’ non-technical skills in the operatingroom: Reliability testing of the NOTSS behavior rating system. World J Surg. 2008;32(4):548–56.
3. Martin JA, Regehr G, Reznick R, Macrae H, Murnaghan J, Hutchison C, et al. Objective structured assessment oftechnical skill (OSATS) for surgical residents. BJS. 1997 Feb 1;84(2):273–8.

