# Correction: Meeting Abstracts for the Society for Simulation in Europe 2024

**DOI:** 10.1186/s41077-024-00312-4

**Published:** 2024-10-07

**Correction** **:** **Advances in Simulation 9, 22 (2024)**

**https://doi.org/10.1186/s41077-024-00287-2**

Manuel López-Baamonde was mistakenly left out of the authorship of the O1 Abstract of the original supplement article. Dr López-Baamonde has since been restored to the authorship.

